# A human iPSC-array-based GWAS identifies a virus susceptibility locus in the NDUFA4 gene and functional variants

**DOI:** 10.1016/j.stem.2022.09.008

**Published:** 2022-10-06

**Authors:** Yuling Han, Lei Tan, Ting Zhou, Liuliu Yang, Lucia Carrau, Lauretta A. Lacko, Mohsan Saeed, Jiajun Zhu, Zeping Zhao, Benjamin E. Nilsson-Payant, Filipe Tenorio Lira Neto, Clare Cahir, Alice Maria Giani, Jin Chou Chai, Yang Li, Xue Dong, Dorota Moroziewicz, The NYSCF Global Stem Cell Array Team, Daniel Paull, Tuo Zhang, Soyeon Koo, Christina Tan, Ron Danziger, Qian Ba, Lingling Feng, Zhengming Chen, Aaron Zhong, Gilbert J. Wise, Jenny Z. Xiang, Hui Wang, Robert E. Schwartz, Benjamin R. tenOever, Scott A. Noggle, Charles M. Rice, Qibin Qi, Todd Evans, Shuibing Chen

**Affiliations:** 1 Department of Surgery, Weill Cornell Medical College, 1300 York Avenue, New York, NY 10065, USA; 2 Center for Energy Metabolism and Reproduction, Institute of Biomedicine and Biotechnology, Shenzhen Institute of Advanced Technology, Chinese Academy of Sciences, Shenzhen, China; 3 Stem Cell Research Facility, Memorial Sloan Kettering Cancer Center, 1275 York Avenue, New York, NY 10065, USA; 4 Department of Microbiology, New York University, 430 E 29th Street, New York, NY 10016, USA; 5 Laboratory of Virology and Infectious Disease, The Rockefeller University, New York, NY 10065, USA; 6 Department of Biochemistry, Boston University School of Medicine, Boston, MA 02118, USA; 7 National Emerging Infectious Diseases Laboratories (NEIDL), Boston University, Boston, MA 02118, USA; 8 Department of Urology, Instituto de Medicina Integral Prof. Fernando Figueira, Recife, Brazil; 9 The Tri-Institutional PhD Program in Chemical Biology, New York, NY, USA; 10 Department of Epidemiology & Population Health, Albert Einstein College of Medicine, 1300 Morris Park Avenue, Bronx, NY 10461, USA; 11 The New York Stem Cell Foundation Research Institute, 619 West 54th Street, 3rd Floor, New York, NY 10019, USA; 12 Genomic Resource Core Facility, Weill Cornell Medical College, New York, NY 10065, USA; 13 Weill Cornell Neuroscience PhD Program, New York, NY, USA; 14 School of Public Health, Shanghai Jiao Tong University School of Medicine, Shanghai, China; 15 Key Laboratory of Pesticide and Chemical Biology (CCNU), Ministry of Education, College of Chemistry, Central China Normal University, Wuhan, Hubei 430079, China; 16 Department of Population Health Sciences, Weill Cornell Medicine, 1300 York Avenue, New York, NY 10065, USA; 17 Department of Urology, Weill Cornell Medicine, 1300 York Avenue, New York, NY 10065, USA; 18 Division of Gastroenterology and Hepatology, Department of Medicine, Weill Cornell Medicine, 1300 York Avenue, New York, NY 10065, USA; 19 Department of Physiology, Biophysics, and Systems Biology, Weill Cornell Medicine, 1300 York Avenue, New York, NY 10065, USA

**Keywords:** NDUFA4, iPSC array, isogenic hiPSC lines, genome-wide association study, single-nucleotide polymorphism, Dengue Virus, SARS-CoV-2, mtDNA, risk allele, type I interferon

## Abstract

Population-based studies to identify disease-associated risk alleles typically require samples from a large number of individuals. Here, we report a human-induced pluripotent stem cell (hiPSC)-based screening strategy to link human genetics with viral infectivity. A genome-wide association study (GWAS) identified a cluster of single-nucleotide polymorphisms (SNPs) in a *cis*-regulatory region of the *NDUFA4* gene, which was associated with susceptibility to Zika virus (ZIKV) infection. Loss of *NDUFA4* led to decreased sensitivity to ZIKV, dengue virus, and SARS-CoV-2 infection. Isogenic hiPSC lines carrying non-risk alleles of SNPs or deletion of the *cis*-regulatory region lower sensitivity to viral infection. Mechanistic studies indicated that loss/reduction of NDUFA4 causes mitochondrial stress, which leads to the leakage of mtDNA and thereby upregulation of type I interferon signaling. This study provides proof-of-principle for the application of iPSC arrays in GWAS and identifies *NDUFA4* as a previously unknown susceptibility locus for viral infection.

## Highlights

• We performed a human iPSC-based genome-wide association study (GWAS)
• This study identified SNPs associated with permissiveness to ZIKV infection
• Loss or reduction of NDUFA4 decreases sensitivity to ZIKV infection
• Loss or reduction of NDUFA4 upregulates type 1 interferon signaling

## Graphical abstract

**Figure:**
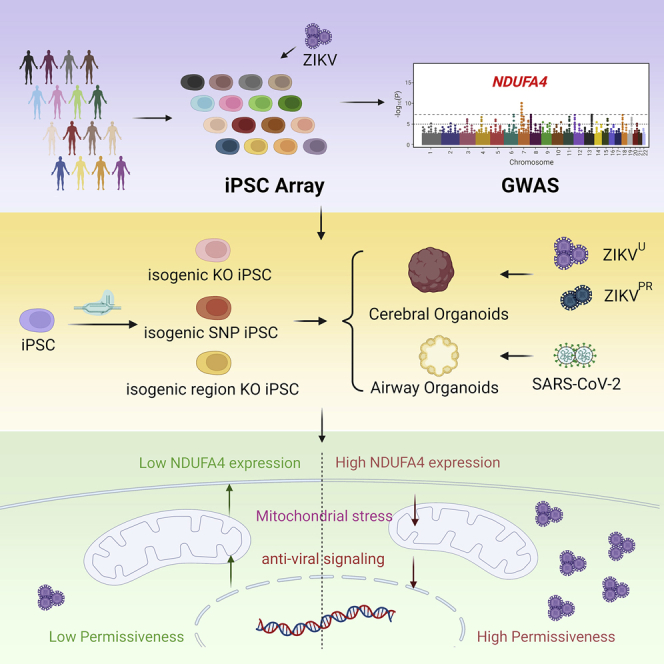

## Teaser

In this study, Han et al. report a proof-of-principle study applying human iPSC arrays in genome-wide association studies and identify NDUFA4 as a previously unknown susceptibility locus for viral infection.

## Introduction

Genome-wide association studies (GWAS) have been broadly applied to identify genetic variants associated with human diseases. Although the sample size that is necessary to achieve an adequate statistical power depends on the genetic architecture of the analyzed trait, GWAS typically require a collection of large samples of individuals, which is expensive and labor-intensive. GWAS are particularly challenging for infectious diseases due to the lack of well-defined cases and controls (Mozzi et al., 2018). Recent GWAS of COVID-19 patients identified a 3p21.31 gene cluster as a genetic susceptibility locus in patients with respiratory failure (Ellinghaus et al., 2020), which is conferred by a genomic segment of around 50 kilobases in size that was inherited from Neanderthals (Zeberg and Pääbo, 2020). GenOMICC (Genetics Of Mortality In Critical Care) identified genetic variants on chromosomal regions 12q24.13 (rs10735079), 19p13.2 (rs74956615), 19p13.3 (rs2109069), and 21q22.1 (rs2236757) that were associated with critical illness in COVID-19 patients (Pairo-Castineira et al., 2021). However, it is not clear whether these variants/gene clusters are mainly involved in viral life cycle or host tissue/organ damage.

Compared with the complexity inherent in human populations, a well-controlled viral infection in a laboratory setting could provide a robust strategy to monitor pathogen susceptibility. For example, a cellular platform was developed to assay *in vitro* phenotypes of infection in genotyped lymphoblastoid cell lines (Salinas et al., 2014; Wang et al., 2018). However, with Epstein-Barr virus transformed lymphoblastoid cell lines, potential genome instability of transformed cell lines might mask or exaggerate an individual’s genetic variation. Induced pluripotent stem cells (iPSCs), which stably carry genetic information of the original donors, provide a valuable platform to study the role of genetic factors in disease progression. Here, we developed a human iPSC (hiPSC) array and performed an unbiased GWAS, which associated the NDUFA4 locus with susceptibility to ZIKV infection.

NDUFA4 is a mitochondrial protein, previously considered a constituent of NADH dehydrogenase. Recent studies showed that NDUFA4 might be a component of cytochrome *c* oxidase, which is a critical member of the mitochondrial electron transport chain (Balsa et al., 2012; Kadenbach, 2017). NDUFA4 mutations have been associated with human neurological disease (Pitceathly et al., 2013). In addition, NDUFA4 expression in clear cell renal cell carcinoma is predictive for cancer-specific survival (Müller et al., 2015). Rearrangement of the NDUFA4 region was also shown to be associated with Tourette syndrome and obsessive-compulsive disorder (Hooper et al., 2012). Using paired isogenic hiPSC lines, we found that loss of *NDUFA4,* knockin of non-risk-allele of two SNPs (rs917172 and rs12386620), or removal of the *cis*-regulatory region led to decreased sensitivity to ZIKV infection and reduced viral replication. In addition, loss/reduction of *NDUFA4* is associated with low permissiveness to other viruses, including dengue virus (DENV) and severe acute respiratory syndrome coronavirus 2 (SARS-CoV-2).

## Results

### Different iPSC lines from an array show variable permissiveness to ZIKV infection

**Figure 1:**
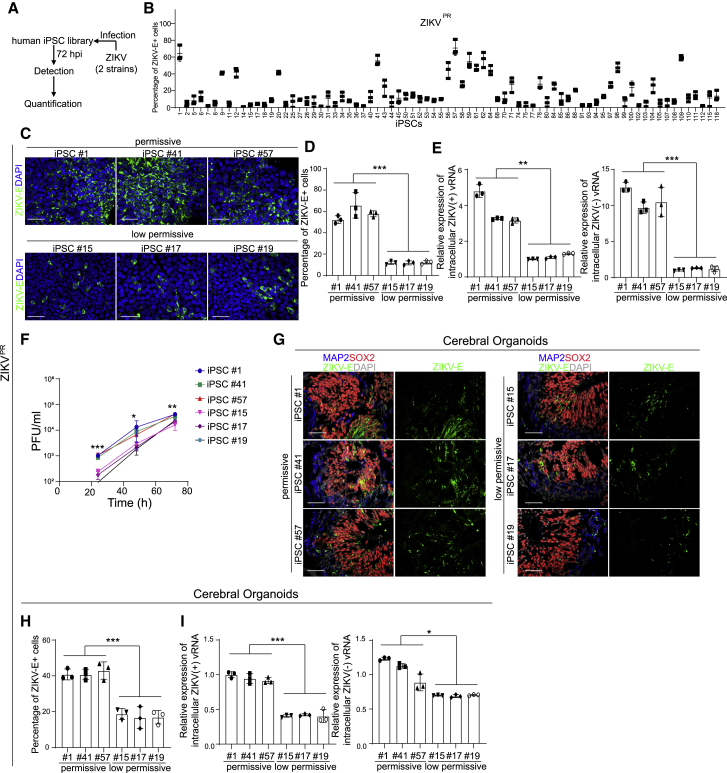
An iPSC-array-based screen of ZIKV infection (A) Scheme of the iPSC screening. (B) The percentage of ZIKV-E positive cells of all iPSC lines upon ZIKV^PR^ infection (ZIKV^PR^, Multiplicity of infection [MOI] = 1). Three biological replicates (each replicate includes one well of 96-well plate) were used to calculate the infectivity. (C and D) Representative confocal images (C) and the quantification (D) of ZIKV-E staining of permissive cell lines iPSC #1, iPSC #41, and iPSC #57 and low-permissive cell lines iPSC #15, iPSC #17, and iPSC #19 at 72 hpi (ZIKV^PR^, MOI = 1). Scale bar, 50 μm. (E) qRT-PCR analysis of (+) and (−) ZIKV vRNA strands of permissive cell lines iPSC #1, iPSC #41, and iPSC #57 and low-permissive cell lines iPSC #15, iPSC #17, and iPSC #19 at 72 hpi (ZIKV^PR^, MOI = 1). The data was normalized to actin beta (*ACTB)*. (F) Multiple step growth curve of ZIKV in the supernatant of permissive cell lines iPSC #1, iPSC #41, and iPSC #57 and low-permissive cell lines iPSC #15, iPSC #17, and iPSC #19 (ZIKV^PR^, MOI = 1). (G and H) Representative confocal images (G) and the quantification (H) of ZIKV-E staining in cerebral organoids derived from permissive cell lines iPSC #1, iPSC #41, and iPSC #57 and low-permissive cell lines iPSC #15, iPSC #17, and iPSC #19 (ZIKV^PR^, 3 × 10^6^ plaque-forming unit [PFU]/mL). Cerebral organoids were age-matched and collected at day 20, then infected with ZIKV for 24 h. After removal of virus-containing medium, organoids were maintained in organoid medium for an additional 3 days. Scale bar, 50 μm. (I) qRT-PCR analysis of (+) and (−) ZIKV vRNA strands in cerebral organoids derived from permissive cell lines iPSC #1, iPSC #41, and iPSC #57 and low-permissive cell lines iPSC #15, iPSC #17, and iPSC #19 (ZIKV^PR^, 3 × 10^6^ PFU/mL). Cerebral organoids were age-matched and collected at day 20, then infected with ZIKV for 24 h. After removal of virus-containing medium, organoids were maintained in organoid medium for an additional 3 days. The value was normalized to *ACTB*. Data are representative of at least three independent experiments. Data are shown as mean ± SD. p values were calculated by unpaired two-tailed Student’s t test; ^∗^p < 0.05, ^∗∗^p < 0.01, and ^∗∗∗^p < 0.001. See also Figure S1.

To perform the screen, a human iPSC array (Paull et al., 2015) containing iPSC lines derived from 77 individuals of varied ethnic backgrounds (Table S1) was infected with either of two ZIKV strains, ZIKV^PR^ (Puerto Rico strain, PRVABC59) or ZIKV^U^ (West Africa strain, MR766) (Figure 1A) (Gabriel et al., 2017; Mlakar et al., 2016; Tang et al., 2016). Immunocytochemistry analysis at 72 h post infection (hpi) revealed that the iPSC lines were differentially sensitive to ZIKV infection (Figures 1B, S1A, and S1B). We normalized the data as the fold change, which is calculated by dividing the percentage of ZIKV envelope protein (ZIKV-E)^+^ cells of each iPSC line by the percentage of ZIKV-E^+^ cells of the iPSC line showing the lowest infection (lowest permissive line). Among 77 iPSC lines, 39 lines were defined as “low permissive” lines upon both ZIKV^PR^ and ZIKV^U^ infection conditions (red line, Figure S1B), and the other lines were defined as “permissive” lines. For example, following equivalent exposure to the virus, iPSC lines #1, #41, and #57 showed a higher percentage of ZIKV E^+^ cells compared to iPSC lines #15, #17, and #19 (ZIKV^PR^: Figures 1C and 1D; ZIKV^U^: Figures S1C and S1D). At the RNA level, much higher levels of both (+) and (−) strands of ZIKV viral RNA (vRNA) were detected in iPSC lines #1, #41, and #57 (ZIKV^PR^: Figure 1E; ZIKV^U^: Figure S1E). Furthermore, a higher yield of infectious ZIKV was detected in the supernatant of iPSC lines #1, #41, and #57 compared to iPSC lines #15, #17, or #19 (ZIKV^PR^: Figure 1F; ZIKV^U^: Figure S1F).

The iPSC lines #1, #15, #17, #19, #41, and #57 were further differentiated toward cerebral organoids that contain neural progenitor cells associated with ZIKV-induced microcephalic phenotypes (Qian et al., 2016; Tang et al., 2016; Zhou et al., 2017). We monitored the ZIKV infection in iPSC-derived cerebral organoids. Both a higher percentage of ZIKV-E^+^ cells (ZIKV^PR^: Figures 1G and 1H) and higher levels of (+) and (−) strands of ZIKV vRNA (ZIKV^PR^: Figure 1I) were detected in cerebral organoids derived from iPSC #1, #41, and #57 than in cerebral organoids derived from iPSC #15, #17, and #19. Together, these data suggest that iPSCs and iPSC-derived cerebral organoids are equally permissive to ZIKV infection, providing validity to the use of iPSC arrays for identifying genetic variants associated with ZIKV infection.

### iPSC-based GWAS identifies SNPs associated with permissiveness to ZIKV infection

**Figure 2:**
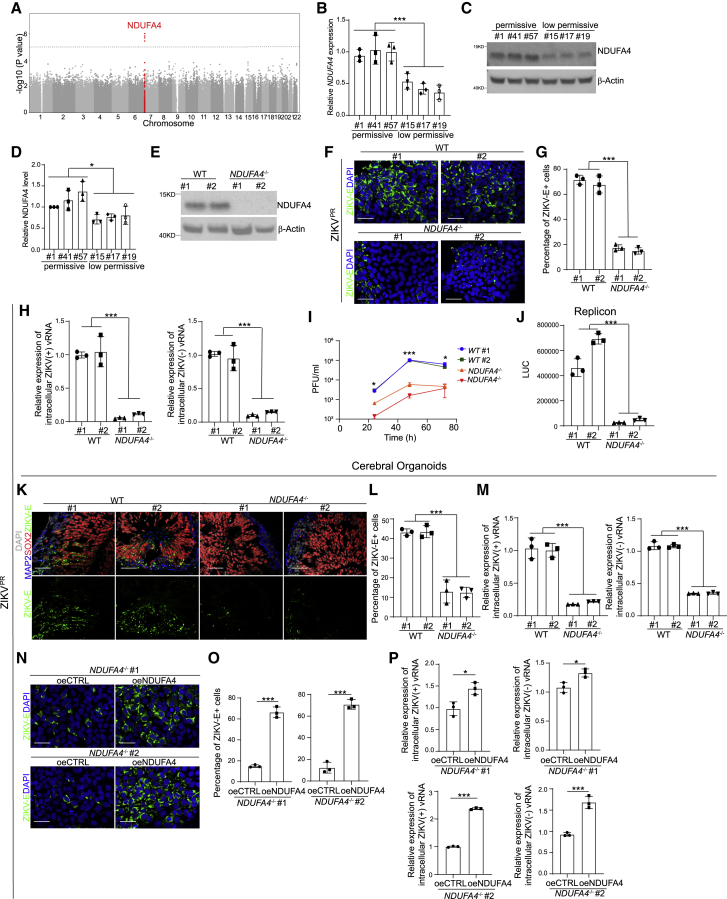
Loss or reduction of NDUFA4 impairs ZIKV replication *in vitro* and *in vivo* (A) Manhattan plot with highlighted risk locus. (B) qRT-PCR analysis of *NDUFA4* mRNA expression levels in permissive iPSC lines iPSC #1, iPSC #41, and iPSC #57 and low-permissive cell lines iPSC #15, iPSC #17, and iPSC #19. The value was normalized to *ACTB*. (C and D) Western blotting analysis (C) and the quantification (D) of NDUFA4 protein expression levels in permissive iPSC lines iPSC #1, iPSC #41, and iPSC #57 and low-permissive cell lines iPSC #15, iPSC #17, and iPSC #19. β-Actin was used as a loading control. (E) Western blotting analysis of NDUFA4 expression levels in WT or *NDUFA4*^*−/−*^ hiPSCs. β-Actin was used as a loading control. (F and G) Representative confocal images (F) and the quantification (G) of ZIKV-E staining in ZIKV-infected WT or *NDUFA4*^*−/−*^ hiPSCs at 72 hpi (ZIKV^PR^, MOI = 1). Scale bar, 50 μm. (H) qRT-PCR analysis of (+) and (−) ZIKV vRNA strands in ZIKV-infected WT or *NDUFA4*^*−/−*^ hiPSCs at 72 hpi (ZIKV^PR^, MOI = 1). The value was normalized to *ACTB*. (I) Multiple step growth curve of ZIKV virus in the supernatant of ZIKV-infected WT or *NDUFA4*^*−/−*^ hiPSCs (ZIKV^PR^, MOI = 1). (J) Luciferase activity of WT or *NDUFA4*^*−/−*^ hiPSCs at 24 h after transfection with ZIKV replicon. (K and L) Representative confocal images (K) and the quantification (L) of ZIKV-E staining in cerebral organoids derived from WT or *NDUFA4*^*−/−*^ hiPSCs (ZIKV^PR^, 3 × 10^6^ PFU/mL). Cerebral organoids were age-matched and collected at day 20, then infected with ZIKV for 24 h. After removal of virus-containing medium, organoids were maintained in organoid medium for an additional 3 days. Scale bar, 50 μm. (M) qRT-PCR analysis of (+) and (−) ZIKV vRNA strands in cerebral organoids derived from WT or *NDUFA4*^*−/−*^ hiPSCs at 72 hpi (ZIKV^PR^, 3 × 10^6^ PFU/mL). Cerebral organoids were age-matched and collected at day 20, then infected with ZIKV for 24 h. After removal of virus-containing medium, organoids were maintained in organoid medium for an additional 3 days. The value was normalized to *ACTB*. (N and O) Representative images (N) and the quantification (O) of ZIKV-E staining in ZIKV-infected *NDUFA4*^*−/−*^-oeCTRL and *NDUFA4*^*−/−*^-oeNDUFA4 hiPSCs at 72 hpi (ZIKV^PR^, MOI = 1). Scale bar, 50 μm. (P) qRT-PCR analysis of (+) and (−) ZIKV vRNA strands in ZIKV-infected *NDUFA4*^*−/−*^-oeCTRL and *NDUFA4*^*−/−*^-oeNDUFA4 hiPSCs at 72 hpi (ZIKV^PR^, MOI = 1). The value was normalized to *ACTB*. Data are representative of at least three independent experiments. Data are shown as mean ± SD. For comparison of permissive and low-permissive lines*,* p values were calculated by unpaired two-tailed Student’s t test. For comparison of WT and KO lines*,* p values were calculated by two-way ANOVA analysis. For comparison of control and overexpression groups, p values were calculated by unpaired two-tailed Student’s t test; ^∗^p < 0.05, ^∗∗∗^p < 0.001. See also Figure S2.

To explore genotype-to-phenotype association, genomic DNA samples from each of the iPSC lines were analyzed using Affymetrix arrays to genotype 691,822 SNPs across the human genome. The associations of SNPs with permissive index was then examined by logistic regression with binary outcomes (low permissive lines versus permissive lines) (Gibson, 2010) (Figure 2A). The top 10 SNPs, which were in strong linkage disequilibrium (r^2^ > 0.8) (Figure 2A and Table S2), were clustered in a ∼6 kb region on human chromosome 7. Among coding and non-coding RNAs located within 1 Mb of this region, NDUFA4 is the only gene expressed in hiPSCs (Table S3). *NDUFA4* encodes NADH dehydrogenase (ubiquinone) 1 alpha subcomplex subunit 4, which is reported to be a mitochondrial protein (Balsa et al., 2012; Pitceathly et al., 2013; Zong et al., 2018). qRT-PCR (Figure 2B) and western blotting assays (Figures 2C and 2D) showed that NDUFA4 expression was significantly lower in the low permissive iPSC lines #15, #17, and #19 compared to the permissive iPSC lines #1, #41, and #57 at both the mRNA and protein levels. No significant difference was detected when comparing the infection rate between cell lines representing different genders (Figure S1G) or ethnic groups (Figure S1H).

To confirm that the differences in ZIKV permissiveness between iPSC lines were due to different levels of NDUFA4 expression, NDUFA4 was overexpressed in low-permissive iPSC lines #15 and #19. qRT-PCR (Figure S1I) and western blotting assays (Figures S1J and S1K) confirmed the increased expression of NDUFA4 at the mRNA and protein levels, respectively. Following infection with ZIKV, a significantly higher percentage of ZIKV-E^+^ cells (ZIKV^PR^: Figures S1L and S1M; ZIKV^U^: Figures S1O and S1P) and increased levels of (+) and (−) strands of ZIKV vRNA (ZIKV^PR^: Figure S1N; ZIKV^U^: Figure S1Q) were detected in iPSCs overexpressing NDUFA4.

### Loss or reduction of NDUFA4 leads to decreased sensitivity to ZIKV infection

**Figure 3:**
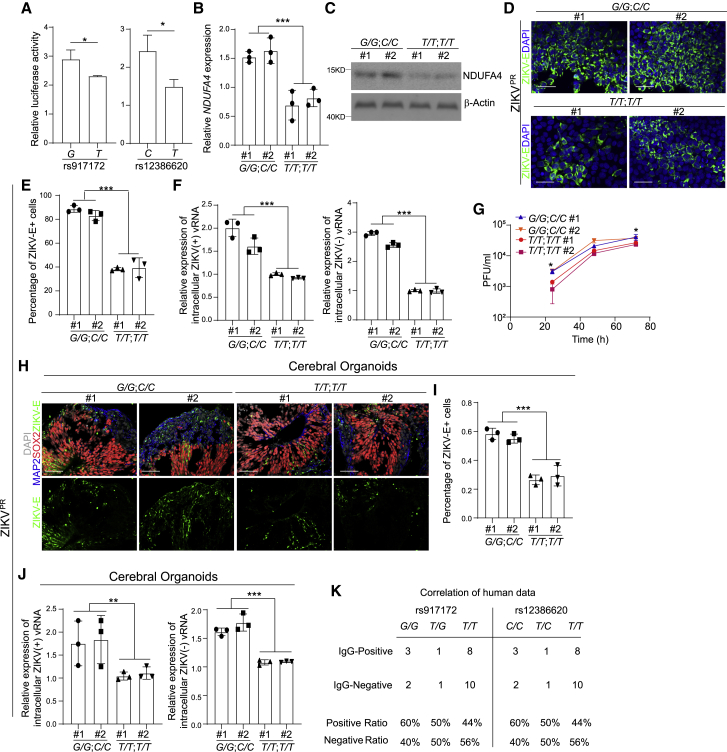
Risk alleles of rs917172 and rs12386620 cause increased sensitivity to ZIKV infection (A) Relative luciferase activity of rs917172 (risk: *G*, non-risk: *T*) and rs12386620 (risk: *C*, non-risk: *T*) in 293T cells. (B) Relative *NDUFA4* mRNA expression in hiPSCs carrying risk (*G/G*; *C/C*) and non-risk (*T/T*; *T/T*) alleles. The value was normalized to *ACTB*. (C) Western blotting analysis of NDUFA4 protein expression levels in hiPSCs carrying risk (*G/G*; *C/C*) and non-risk (*T/T*; *T/T*) alleles. β-Actin was used as a loading control. (D and E) Representative confocal images (D) and the quantification (E) of ZIKV-E staining in ZIKV-infected hiPSCs carrying risk (*G/G*; *C/C*) and non-risk (*T/T*; *T/T*) alleles at 72 hpi (ZIKV^PR^, MOI = 1). Scale bar, 50 μm. (F) qRT-PCR analysis of (+) and (−) ZIKV vRNA strands in ZIKV-infected hiPSCs carrying risk (*G/G*; *C/C*) and non-risk (*T/T*; *T/T*) alleles at 72 hpi (ZIKV^PR^, MOI = 1). The value was normalized to *ACTB*. (G) Multiple step growth curve of ZIKV in the supernatant of ZIKV-infected hiPSCs carrying risk (*G/G*; *C/C*) and non-risk (*T/T*; *T/T*) alleles (ZIKV^PR^, MOI = 1). (H and I) Representative confocal images (H) and the quantification (I) of ZIKV-E staining in cerebral organoids derived from hiPSCs carrying risk (*G/G*; *C/C*) and non-risk (*T/T*; *T/T*) alleles (ZIKV^PR^, 3 × 10^6^ PFU/mL). Cerebral organoids were age-matched and collected at day 20, then infected with ZIKV for 24 h. After removal of virus-containing medium, organoids were maintained in organoid medium for an additional 3 days. Scale bar = 50 μm. (J) qRT-PCR analysis of (+) and (−) ZIKV vRNA strands of cerebral organoids derived from hiPSCs carrying risk (*G/G*; *C/C*) and non-risk (*T/T*; *T/T*) alleles (ZIKV^PR^, 3 × 10^6^ PFU/mL). Cerebral organoids were age-matched and collected at day 20, then infected with ZIKV for 24 h. After removal of virus-containing medium, organoids were maintained in organoid medium for an additional 3 days. The value was normalized to *ACTB*. (K) The percentage of IgG^+^ or IgM^+^ patients carrying risk or non-risk allele of rs917172 and rs12386620. Data are representative of at least three independent experiments. Data are shown as mean ± SD. For comparison with more than two samples*,* p values were calculated by two-way ANOVA analysis. For comparison with two samples*,* p values were calculated by unpaired two-tailed Student’s t test; ^∗^p < 0.05, ^∗∗^p < 0.01 and ^∗∗∗^p < 0.001. See also Figure S3.

**Figure 4:**
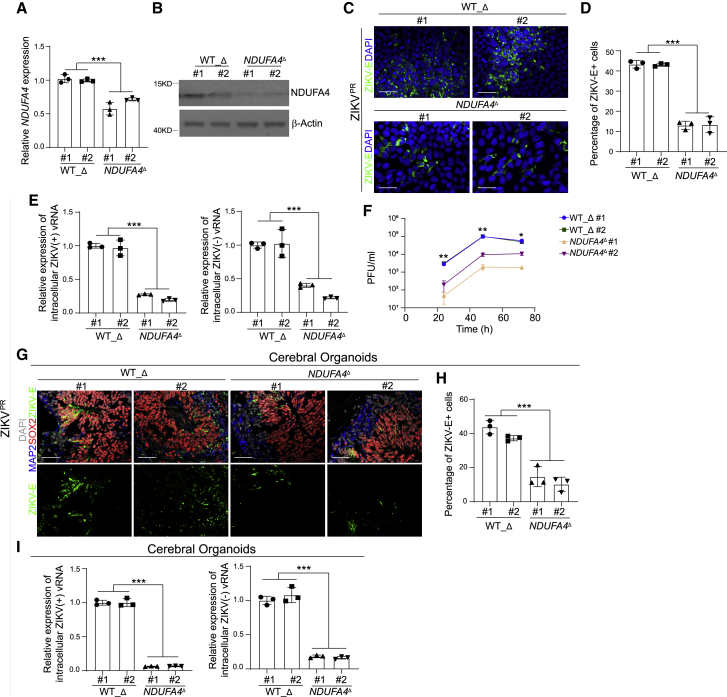
Deletion of a *cis*-regulatory region of *NDUFA4* decreased the infection of ZIKV infection (A) qRT-PCR analysis of *NDUFA4* mRNA expression levels in WT_Δ and *NDUFA4*^Δ^ hiPSCs. The value was normalized to *ACTB*. (B) Western blotting analysis of NDUFA4 protein expression levels in WT_Δ and *NDUFA4*^Δ^ hiPSCs. β-Actin was used as a loading control. (C and D) Representative confocal images (C) and the quantification (D) of ZIKV-E staining in ZIKV-infected WT_Δ and *NDUFA4*^Δ^ hiPSCs at 72 hpi (ZIKV^PR^, MOI = 1). Scale bar, 50 μm. (E) qRT-PCR analysis of (+) and (−) ZIKV vRNA strands in ZIKV-infected WT_Δ and *NDUFA4*^Δ^ hiPSCs at 72 hpi (ZIKV^PR^, MOI = 1). The value was normalized to *ACTB*. (F) Multiple step growth curve of ZIKV in the supernatant of ZIKV-infected WT_Δ and *NDUFA4*^Δ^ hiPSCs (ZIKV^PR^, MOI = 1). (G and H) Representative images (G) and the quantification (H) of ZIKV-E staining in cerebral organoids derived from WT_Δ and *NDUFA4*^Δ^ hiPSCs (ZIKV^PR^, 3 × 10^6^ PFU/mL). Cerebral organoids were age-matched and collected at day 20, then infected with ZIKV for 24 h. After removal of virus-containing medium, organoids were maintained in organoid medium for an additional 3 days. Scale bar, 50 μm. (I) qRT-PCR analysis of (+) and (−) ZIKV vRNA strands in cerebral organoids derived from WT_Δ and *NDUFA4*^Δ^ hiPSCs (ZIKV^PR^, 3 × 10^6^ PFU/mL). Cerebral organoids were age-matched and collected at day 20, then infected with ZIKV for 24 h. After removal of virus-containing medium, organoids were maintained in organoid medium for an additional 3 days. The value was normalized to *ACTB*. Data are representative of at least three independent experiments. Data are shown as mean ± SD. p values were calculated by two-way ANOVA analysis; ^∗^p < 0.05, ^∗∗^p < 0.01 and ^∗∗∗^p < 0.001. See also Figure S4.

**Figure 5:**
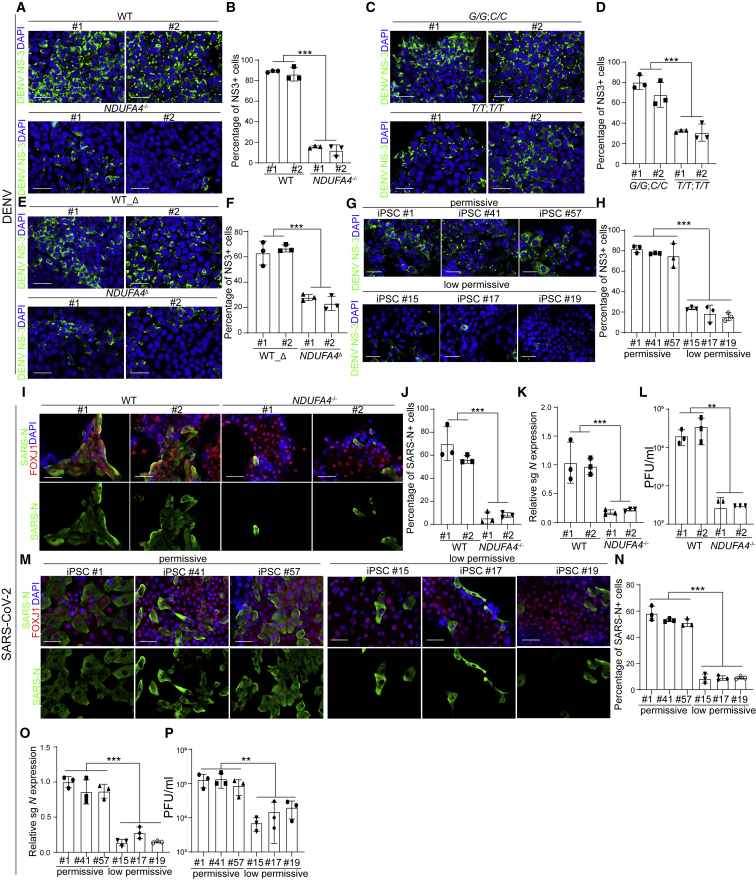
NDUFA4 is associated with DENV and SARS-CoV-2 infection (A and B) Representative confocal images (A) and the quantification (B) of DENV NS3 staining in ZIKV-infected WT or *NDUFA4*^*−/−*^ hiPSCs at 72 hpi (DENV, MOI = 1). Scale bar, 50 μm. (C and D) Representative confocal images (C) and the quantification (D) of DENV NS3 staining in hiPSCs carrying risk (*G/G*; *C/C*) or non-risk (*T/T*; *T/T*) alleles at 72 hpi (DENV, MOI = 1). Scale bar, 50 μm. (E and F) Representative confocal images (E) and the quantification (F) of DENV NS3 staining in DENV-infected WT_Δ or *NDUFA4*^Δ^ hiPSCs at 72 hpi (DENV, MOI = 1). Scale bar, 50 μm. (G and H) Representative confocal images (G) and the quantification (H) of DENV NS3 staining in DENV-infected permissive cell lines iPSC #1, iPSC #41, and iPSC #57 or low-permissive cell lines iPSC #15, iPSC #17, and iPSC #19 at 72 hpi (DENV, MOI = 1). Scale bar, 50 μm. (I and J) Representative confocal images (I) and the quantification (J) of SARS-N in FOXJ1^+^ ciliated cells in airway organoids derived from WT or *NDUFA4*^*−/−*^ hiPSCs at 24 hpi (SARS-CoV-2, MOI = 0.1). (K) Relative expression of viral subgenomic RNA (N) transcription in SARS-CoV-2-infected airway organoids derived from WT or *NDUFA4*^*−/−*^ hiPSCs at 24 hpi (SARS-CoV-2, MOI = 0.1). The value was normalized to *ACTB*. (L) Viral titers of SARS-CoV-2 infected airway organoids derived from WT or *NDUFA4*^*−/−*^ hiPSCs at 24 hpi (SARS-CoV-2, MOI = 0.05). (M and N) Representative confocal images (M) and the quantification (N) of SARS-N in FOXJ1^+^ ciliated cells in airway organoids derived from permissive cell lines iPSC #1, iPSC #41, or iPSC #57 and low-permissive cell lines iPSC #15, iPSC #17, and iPSC #19 at 24 hpi (SARS-CoV-2, MOI = 0.1). (O) Relative expression level of viral subgenomic RNA (N) transcripts in SARS-CoV-2-infected airway organoids derived from permissive cell lines iPSC #1, iPSC #41, and iPSC #57 or low-permissive cell lines: iPSC #15, iPSC #17, and iPSC #19 at 24 hpi (SARS-CoV-2, MOI = 0.1). The value was normalized to *ACTB*. (P) Viral titers of SARS-CoV-2-infected airway organoids derived from permissive cell lines iPSC #1, iPSC #41, and iPSC #57 or low-permissive cell lines iPSC #15, iPSC #17, and iPSC #19 at 24 hpi (SARS-CoV-2, MOI = 0.05). Data are representative of at least three independent experiments. Data are shown as mean ± SD. For permissive and low-permissive cell lines*,* p values were calculated by unpaired two-tailed Student’s t test. For other figure panels, p values were calculated by two-way ANOVA analysis; ^∗∗^p < 0.01, and ^∗∗∗^p < 0.001. See also Figure S5.

**Figure 6:**
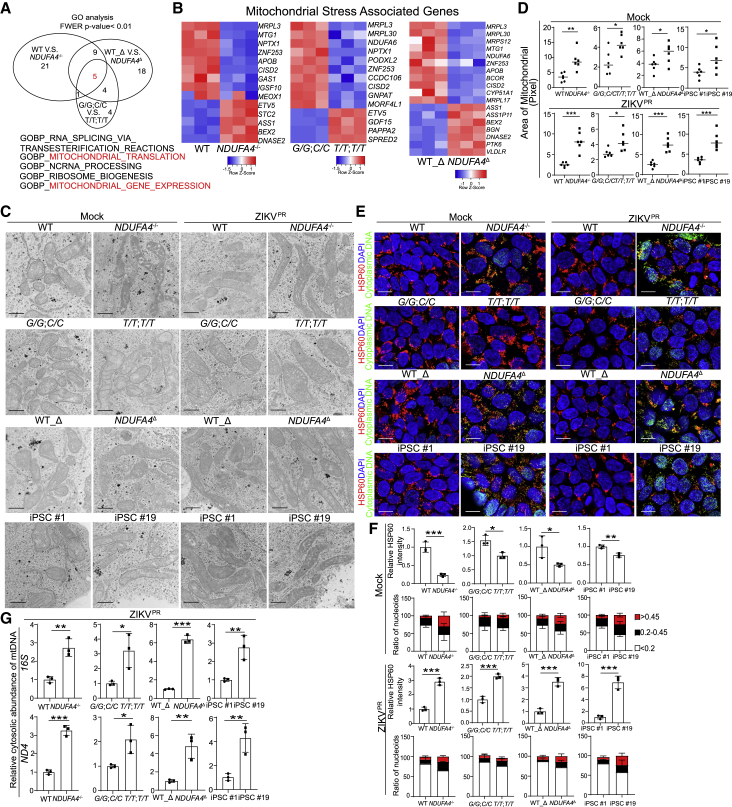
Loss or reduction of NDUFA4 causes mitochondrial DNA leakage (A) Correlation of pathways analyzed by Gene Ontology analysis of hiPSCs. Five pathways are enriched in all 3 groups: WT versus *NDUFA4*^*−/−*^, risk (*G/G*; *C/C*) versus non-risk (*T/T*; *T/T*), and WT_Δ versus *NDUFA4*^Δ^. (B) Heatmap of mitochondrial stress signature genes in 3 groups of hiPSCs. 3 groups: WT versus *NDUFA4*^*−/−*^, risk (*G/G*; *C/C*) versus non-risk (*T/T*; *T/T*), and WT_Δ versus *NDUFA4*^Δ^. (C and D) Representative electron microscopy images (C) and the quantification of mitochondrial size (D) of iPSC lines with mock or ZIKV infection at 48 hpi (ZIKV^PR^, MOI = 1). 4 groups: WT versus *NDUFA4*^*−/−*^, risk (*G/G*; *C/C*) versus non-risk (*T/T*; *T/T*), WT_Δ versus *NDUFA4*^Δ^, and iPSC #1 versus iPSC #19. Scale bars, 500 nm. (E) Representative confocal images of hiPSCs at 48 hpi with mock or ZIKV infection stained with anti-HSP60 or anti-DNA antibodies (ZIKV^PR^, MOI = 1). 4 groups: WT versus *NDUFA4*^*−/−*^, risk (*G/G*; *C/C*) versus non-risk (*T/T*; *T/T*), WT_Δ versus *NDUFA4*^Δ^, and iPSC #1 versus iPSC #19. Scale bar, 10 μm. (F) Quantification of HSP60 staining intensity and nucleoid area in (E) in mock or infected conditions (ZIKV^PR^, MOI = 1). (G) qPCR analysis of mitochondrial DNA leakage in cytoplasm after ZIKV infection in hiPSCs at 48 hpi (ZIKV^PR^, MOI = 1). 4 groups: WT versus *NDUFA4*^*−/−*^, risk (*G/G*; *C/C*) versus non-risk (*T/T*; *T/T*), WT_Δ versus *NDUFA4*^Δ^, and iPSC #1 versus iPSC #19. Data are representative of at least three independent experiments. Data are shown as mean ± SD. p values were calculated by unpaired two-tailed Student’s t test; ^∗^p < 0.05, ^∗∗^p < 0.01, and ^∗∗∗^p < 0.001. See also Figure S6.

**Figure 7:**
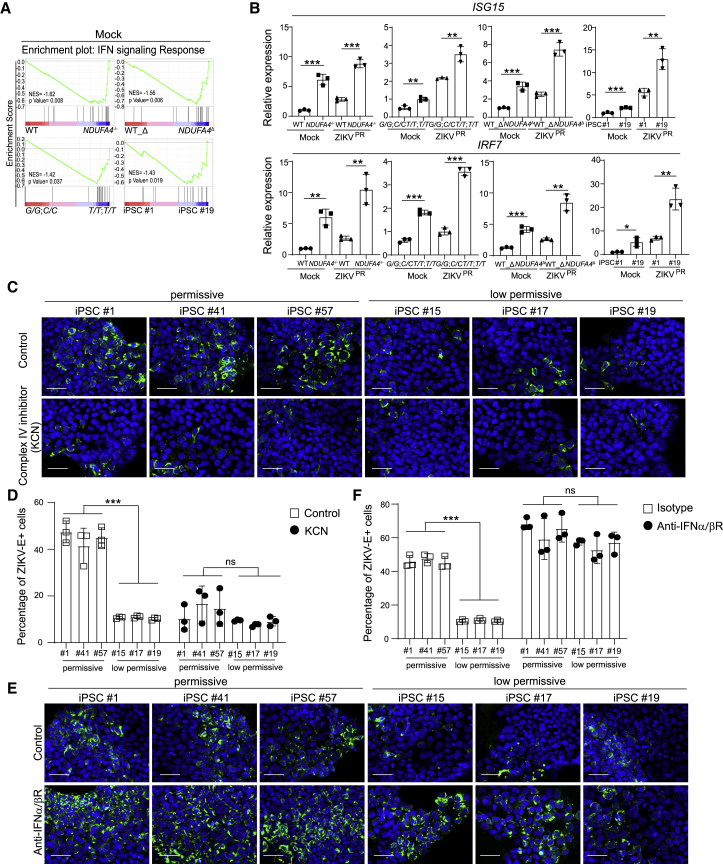
Loss or reduction of NDUFA4 triggers type I interferon signaling (A) Gene Set Enrichment Analysis of type I interferon signal pathway in 4 groups of hiPSCs. 4 groups: WT versus *NDUFA4*^*−/−*^, risk (*G/G*; *C/C*) versus non-risk (*T/T*; *T/T*), WT_Δ versus *NDUFA4*^Δ^, and iPSC #1 versus iPSC #19. (B) qRT-PCR analysis of *ISG15* and *IRF7* mRNA expression levels with mock or ZIKV infection at 72 hpi (ZIKV^PR^, MOI = 1). 4 groups: WT versus *NDUFA4*^*−/−*^, risk (*G/G*; *C/C*) versus non-risk (*T/T*; *T/T*), WT_Δ versus *NDUFA4*^Δ^, and iPSC #1 versus iPSC #19. The value was normalized to *ACTB*. (C and D) Representative confocal images (C) and the quantification (D) of ZIKV-E staining of permissive cell lines iPSC #1, iPSC #41, and iPSC #57 and low-permissive cell lines iPSC #15, iPSC #17, and iPSC #19 treated with potassium cyanide (KCN) at 72 hpi (ZIKV^PR^, MOI = 1). Scale bar, 50 μm. (E and F) Representative confocal images (E) and the quantification (F) of ZIKV-E staining of permissive cell lines iPSC #1, iPSC #41, and iPSC #57 and low-permissive cell lines: iPSC #15, iPSC #17, and iPSC #19 treated with blocking antibodies of IFNAR at 72 hpi (ZIKV^PR^, MOI = 1). Scale bar, 50 μm. Data are representative of at least three independent experiments. Data are shown as mean ± SD. p values were calculated by unpaired two-tailed Student’s t test; ^∗^p < 0.05, ^∗∗^p < 0.01, and ^∗∗∗^p < 0.001. See also Figure S7.

To probe the functional role of NDUFA4 in ZIKV infection, we used a CRISPR-based gene knockout strategy to create *NDUFA4*^*−/−*^ iPSC lines on the background of iPSC #9, which is a moderately permissive parental line (Figure S2A; Table S4). Two independent iPSC lines carrying compound heterozygous mutations were chosen (*NDUFA4*^*−/−*^ #1, *NDUFA4*^*−/−*^ #2). The indel mutations of *NDUFA4*^*−/−*^ iPSC lines were validated with DNA sequencing (Figure S2B). iPSC clones isolated from the targeting procedure that did not harbor *NDUFA4* mutations were used as wild-type (WT) controls (WT #1, WT #2). Western blotting analysis further validated the loss of NDUFA4 expression in *NDUFA4*^*−/−*^ iPSCs (Figure 2E). All WT and *NDUFA4*^*−/−*^ iPSC lines maintained pluripotent-like colony morphology and expression of pluripotency markers NANOG, OCT4, SOX2, SSEA4, TRA-1-60, and TRA-1-81 (Figure S2C), indicating that loss of NDUFA4 does not impact pluripotency. WT and *NDUFA4*^*−/−*^ iPSC lines were infected with either ZIKV^PR^ or ZIKV^U^. *NDUFA4*^*−/−*^ iPSC lines exhibited decreased sensitivity to ZIKV infection compared to the WT lines. The percentage of ZIKV-E^+^ cells was significantly lower in *NDUFA4*^*−/−*^ iPSCs than WT iPSCs at 72 hpi (ZIKV^PR^: Figures 2F and 2G; ZIKV^U^: Figure 1, Figure 2, Figure 3, Figure 4, Figure 5, Figure 6, Figure 7D and S2E). qRT-PCR was used to monitor the intracellular levels of ZIKV (+) and (−) vRNA strands at 72 hpi. Both strands were at significantly lower levels in *NDUFA4*^*−/−*^ iPSCs compared to WT iPSCs (ZIKV^PR^: Figure 2H; ZIKV^U^: Figure S2F). Likewise, the level of infectious virus released into the supernatant of *NDUFA4*^*−/−*^ iPSCs was lower than those of WT iPSCs (ZIKV^PR^: Figure 2I; ZIKV^U^: Figure S2G). *NDUFA4*^*−/−*^ or WT iPSCs were transfected with *in vitro* transcribed ZIKV replicon RNA to determine whether loss of NDUFA4 affects viral RNA replication (Rusanov et al., 2018; Saeed et al., 2015). The luminescence signal in *NDUFA4*^*−/−*^ iPSCs was significantly lower than that in WT cells (Figure 2J). To determine whether loss of NDUFA4 might also affect virus entry, we infected WT and *NDUFA4*^*−/−*^ iPSCs with ZIKV reporter virus particles (RVPs) that enter cells in a ZIKV-envelope-protein-dependent manner (Robbiani et al., 2017). There was no statistical difference in reporter activity between *NDUFA4*^*−/−*^ and WT iPSCs (Figure S2H), suggesting that loss of *NDUFA4* impacts viral RNA replication rather than viral entry.

To determine the role of NDUFA4 in ZIKV infection of differentiated cells, WT and *NDUFA4*^*−/−*^ iPSCs were differentiated toward cerebral organoids. Similar to iPSCs, a lower percentage of ZIKV-E^+^ cells (ZIKV^PR^: Figures 2K and 2L) and less (+) and (−) strand ZIKV vRNA (ZIKV^PR^: Figure 2M; ZIKV^U^: Figure S2I) were detected in *NDUFA4*^*−/−*^ iPSC-derived cerebral organoids as compared to WT iPSC-derived cerebral organoids. There was no significant difference regarding the percentage of Ki67^+^ cells in SOX2^+^ cells in WT and *NDUFA4*^*−/−*^ iPSC-derived cerebral organoids (Figures S2J and S2K), suggesting that loss of NDUFA4 does not affect proliferation of neural progenitor cells.

To confirm that the low permissiveness of *NDUFA4*^*−/−*^ iPSCs was due to the loss of NDUFA4, NDUFA4 was overexpressed in *NDUFA4*^*−/−*^ iPSC lines, which was validated by western blotting (Figure S2L). Following infection, the *NDUFA4*^*−/−*^ iPSC lines overexpressing NDUFA4 showed a higher percentage of ZIKV-E^+^ cells (ZIKV^PR^: Figures 2N and 2O; ZIKV^U^: Figures S2M and S2N) and higher level of ZIKV (+) and (−) vRNA strands (ZIKV^PR^: Figure 2P; ZIKV^U^: Figure S2O) at 72 hpi compared to *NDUFA4*^*−/−*^ iPSC lines.

### Isogenic hiPSC lines carrying risk alleles rs917172 and rs12386620 show enhanced permissiveness to ZIKV infection

This iPSC-based GWAS identified several SNPs that are associated with permissiveness to ZIKV infection. To determine the impact of risk/non-risk alleles of these variants, we cloned approximately ∼140–250 bp long regions around these variants (Table S4) in a reporter construct containing miniCMV:Luc to evaluate regulatory activity. Corresponding pairs of reporters carrying non-risk/risk alleles, respectively, were created by site-directed mutagenesis. The constructs containing non-risk and risk alleles were transfected into 293T cells and monitored for luciferase activity (Figures 3A and S3A). A significant difference of luciferase activity was detected between risk and non-risk alleles representing four variants, including rs917172, rs12386620, rs6958970, and rs6954962. Due to the inherent complexity of studying isogenic lines carrying multiple mutations, we focused on the two variants that showed the lowest p value in GWAS, rs917172 and rs12386620. Since rs917172 and rs12386620 showed high linkage disequilibrium, CRISPR-based gene editing was applied to induce both the risk *G* allele for rs917172 and the risk *C* allele for rs12386620 (Figure S3B). Two clones that carried non-risk *T* alleles for both rs917172 and rs12386620 loci (Figure S3B) were used as controls. qRT-PCR (Figure 3B) and western blotting (Figure 3C) assays validated the increased NDUFA4 expression in iPSC lines carrying risk alleles (*G/G*; *C/C*) at the mRNA and protein levels, respectively. The isogenic iPSC lines carrying risk alleles (*G/G*; *C/C*) and non-risk alleles (*T/T*; *T/T*) were infected by ZIKV. The percentage of ZIKV-E^+^ cells was higher in iPSC lines carrying risk alleles (*G/G*; *C/C*) than iPSC lines carrying non-risk alleles (*T/T*; *T/T*) (ZIKV^PR^: Figures 3D and 3E; ZIKV^U^: Figures S3C and S3D). Likewise, the level of ZIKV (+) and (−) vRNA strands (ZIKV^PR^: Figure 3F; ZIKV^U^: Figure S3E) and the level of infectious virus (ZIKV^PR^: Figure 3G; ZIKV^U^: Figure S3F) were significantly higher in iPSC lines carrying risk alleles (*G/G*; *C/C*) than in iPSC lines carrying non-risk alleles (*T/T*; *T/T*).

To determine the role of genetic variants in disease-relevant cell types, the isogenic iPSC lines carrying risk alleles (*G/G*; *C/C*) and non-risk alleles (*T/T*; *T/T*) were differentiated toward cerebral organoids. Similarly, cerebral organoids derived from iPSC lines carrying risk alleles (*G/G*; *C/C*) showed a higher percentage of ZIKV-E^+^ cells (ZIKV^PR^: Figures 3H and 3I) and increased level of ZIKV (+) and (−) vRNA strands (ZIKV^PR^: Figure 3J; ZIKV^U^: Figure S3G) than those carrying non-risk alleles (*T/T*; *T/T*).

Previous studies showed that human testicular tissue and germ cells are permissive to ZIKV infection (Matusali et al., 2018; Robinson et al., 2018). Here, we analyzed vas deferens and testicular biopsy samples collected from patients with or without a history of ZIKV infection, based on IgG/IgM information (Table S5). Higher positive ZIKV IgG/IgM ratios were detected in patients carrying the risk *G* allele of rs917172 and the risk *C* allele of rs12386620 (Figure 3K).

### Deletion of *cis*-regulatory elements causes decreased sensitivity to ZIKV infection

Ten SNPs associated with the *NDUFA4* gene were located around 0.8 Mb downstream of the *NDUFA4* locus on human chromosome 7 (Figure S4A). Five out of 10 SNPs associated with the *NDUFA4* gene, including rs917172 and rs12386620, were clustered in a ∼1 kb region. To determine whether this region is involved in the regulation of the *NDUFA4* gene, two single guide RNAs (sgRNAs) were designed to knockout this region using CRISPR-Cas9 (Table S4). Two independent lines were identified by PCR of genomic DNA with homozygous knockout of the 1055 bp targeted region, which was validated by DNA sequencing (Figure S4B). Both mutant *NDUFA4*^Δ^ iPSC lines (*NDUFA4*^Δ^ #1, *NDUFA4*^Δ^ #2) were compared with two control WT lines derived from the same targeting procedure that were not mutated (WT_Δ #1, WT_Δ #2). Interestingly, both qRT-PCR (Figure 4A) and western blotting assays (Figure 4B) showed that the *NDUFA4*^Δ^ lines had decreased expression levels of NDUFA4. In addition, *NDUFA4*^Δ^ iPSC lines showed reduced sensitivity to infection of either ZIKV strain (ZIKV^PR^: Figures 4C and 4D; ZIKV^U^: Figures S4C and S4D). Lower levels of (+) and (−) ZIKV vRNA strands were detected in *NDUFA4*^*Δ*^ iPSC lines compared to WT_Δ iPSC lines (ZIKV^PR^: Figure 4E; ZIKV^U^: Figure S4E). Finally, less infectious virus was detected in the supernatant of *NDUFA4*^Δ^ iPSC lines than those of WT_Δ iPSC lines (ZIKV^PR^: Figure 4F; ZIKV^U^: Figure S4F).

*NDUFA4*^Δ^ and WT_Δ iPSC lines were then differentiated into cerebral organoids. Similar to the iPSCs, following infection, cerebral organoids derived from *NDUFA4*^Δ^ iPSC lines showed a lower percentage of ZIKV-E^+^ cells (ZIKV^PR^: Figures 4G and 4H) and reduced levels of ZIKV (+) and (−) vRNA strands (ZIKV^PR^: Figure 4I; ZIKV^U^: Figure S4G) compared to WT_Δ iPSC cerebral organoids. Taken together, these data suggest that the genomic region containing this cluster of SNPs functions as a *cis*-regulatory region for *NDUFA4* expression, which is consistent with higher NDUFA4 levels increasing permissiveness to ZIKV infection.

### NDUFA4 is associated with permissiveness to dengue virus and SARS-CoV-2 infection

Thus far, using the iPSC-array-based screen, we identified the *NDUFA4* gene and showed that its regulatory region and genetic variants are associated with permissiveness to ZIKV infection. To determine whether *NDUFA4* is relevant to infection by other flaviviruses, *NDUFA4*^*−/−*^ or control iPSCs were infected with dengue virus (DENV). The percentages of NS3^+^ cells were significantly lower in *NDUFA4*^*−/−*^ iPSCs than for WT iPSCs (Figures 5A and 5B). Consistent with the results for ZIKV, iPSC lines carrying risk (*G/G*; *C/C*) alleles showed higher permissiveness to DENV than iPSC lines carrying non-risk (*T/T*; *T/T*) alleles (Figures 5C and 5D). In addition, WT_Δ iPSCs showed higher permissiveness to DENV than *NDUFA4*^Δ^ cells (Figures 5E and 5F). Furthermore, iPSC #1, #41, and #57 were also more sensitive to DENV infection than iPSC #15, #17, and #19 (Figures 5G and 5H).

We next examined the relevance of NDUFA4 to a non-flavivirus, SARS-CoV-2, which is the causative agent of the current global pandemic. The WT and *NDUFA4*^*−/−*^ iPSCs were differentiated into lung airway organoids, which we showed previously are permissive to SARS-CoV-2 infection (Duan et al., 2021). The differentiation was confirmed by immunostaining with antibodies directed against airway markers, including E-Cadherin (a pan-epithelial cell marker), FOXJ1 and acetylated α-tubulin (ciliated cell markers), and MUC5AC (a goblet cell marker) (Figure S5A). The iPSC-derived airway organoids were infected with SARS-CoV-2 (USA-WA1/2020). Previous studies have suggested that ACE2, the main receptor of SARS-CoV-2, is highly expressed in airway ciliated cells (Duan et al., 2021; Ziegler et al., 2020). Therefore, we monitored the infection efficiency of SARS-CoV-2 in FOXJ1^+^ ciliated cells and found significantly less SARS-N^+^; FOXJ1^+^ ciliated cells in *NDUFA4*^*−/−*^ iPSC-derived airway organoids than in WT iPSC-derived airway organoids (Figures 5I and 5J). Consistent with this finding, the level of viral subgenomic N RNA, an indicator for the presence of actively replicating virus, was also lower in *NDUFA4*^*−/−*^ iPSC-derived airway organoids than WT iPSC-derived airway organoids (Figure 5K). Also, less infectious virus was detected in the supernatant of *NDUFA4*^*−/−*^ iPSC-derived airway organoids than WT iPSC-derived airway organoids (Figure 5L). In addition, the airway organoids derived from iPSC #1, #41, and #57 showed higher permissiveness to SARS-CoV-2 infection. The percentage of SARS-N^+^; FOXJ1^+^ cells in airway organoids derived from iPSC #1, #41, and #57 was higher than those of airway organoids derived from iPSC #15, #17, and #19 (Figures 5M and 5N). Similarly, much higher levels of viral subgenomic RNA were detected in airway organoids derived from iPSC #1, #41, and #57 than airway organoids derived from iPSC #15, #17, and #19 (Figure 5O). Consistent with this, more infectious virus was detected in the supernatant of airway organoids derived from iPSC #1, #41, and #57 than those derived from iPSC #15, #17, and #19 (Figure 5P). Moreover, a higher level of NDUFA4 expression was detected in lung tissues from COVID-19 patients compared to healthy donors (Figure S5B) (Han et al., 2021), which is consistent with the previous sequencing results from bronchoalveolar lavage fluid (BALF) of COVID-19 patients and healthy controls (Zhou et al., 2020). We also compared the expression of NDUFA4 in severe and mild COVID-19 patients (GSE196822). There is a trend toward higher levels of NDUFA4 expression in severe compared to mild COVID-19 patients (Figure S5C). This data suggests a contributing role for NDUFA4 in COVID-19. However, there are additional factors that can contribute to disease severity, such as age, inflammation, and co-morbidities (Abdulrahman et al., 2021).

### Loss or reduction of NDUFA4 causes mtDNA leakage

NDUFA4 has been reported to be a complex IV subunit of the mammalian electron transport chain (Balsa et al., 2012), and mutations of *NDUFA4* are associated with mitochondrial dysfunction (Yagil et al., 2018). To determine how NDUFA4 might impact viral infection, RNA-sequencing was used to compare the global gene expression profiles in WT and *NDUFA4*^*−/−*^ iPSCs, iPSCs carrying non-risk (*T/T*; *T/T*) and risk (*G/G*; *C/C*) alleles, and WT_Δ and *NDUFA4*^Δ^ iPSCs. Gene Ontology analyses were carried out to examine the gene expression changes in WT versus *NDUFA4*^*−/−*^ iPSCs, iPSCs carrying risk (*G/G*; *C/C*) versus non-risk (*T/T*; *T/T*) alleles, and WT_Δ versus *NDUFA4*^Δ^ iPSCs. Five pathways were found to be commonly perturbated by loss of NDUFA4, genetic variants, and deletion of the *NDUFA4* regulatory region, two of which were mitochondria-related pathways (Figure 6A). A set of signature genes related to mitochondrial stress (Quiros et al., 2017) were also dysregulated in *NDUFA4*^*−/−*^ versus WT iPSCs, iPSCs carrying risk (*G/G*; *C/C*) versus non-risk (*T/T*; *T/T*) alleles, and *NDUFA4*^Δ^ versus WT_Δ iPSCs (Figure 6B).

To further examine the effect of NDUFA4 on mitochondria, we compared the mitochondrial morphology of different isogenic iPSC lines by electron microscopy. Significantly more elongated, interconnected mitochondrial networks consistent with a hyperfused phenotype were observed in *NDUFA4*^*−/−*^ iPSCs versus WT iPSCs, non-risk (*T/T*; *T/T*) versus risk allele (*G/G*; *C/C*) iPSCs, and *NDUFA4*^Δ^ iPSCs versus WT_Δ iPSCs under both mock and ZIKV- or DENV-infected conditions (iPSCs in Mock or ZIKV^PR^ conditions: Figures 6C and 6D; iPSCs in DENV and ZIKV^U^ conditions: Figures S6A and S6B). In addition, elongated mitochondria with a hyperfusion phenotype were also more frequently detected in low-permissive iPSC line #19 but not in permissive iPSC line #1 under both mock and virus-infected conditions (iPSCs in Mock or ZIKV^PR^ conditions: Figures 6C and 6D; iPSCs in DENV and ZIKV^U^ conditions: Figures S6A and S6B). Since mitochondrial stress can cause leakage of mitochondrial (mtDNA), confocal microscopy was used to determine the number and size of mtDNA nucleoids using anti-HSP60 and anti-DNA antibodies (West et al., 2015). There was an increased percentage of large nucleoids (>0.45) and medium nucleoids (0.2–0.45) in *NDUFA4*^*−/−*^ iPSCs versus WT iPSCs, non-risk (*T/T*; *T/T*) versus risk allele (*G/G*; *C/C*) iPSCs, and *NDUFA4*^Δ^ iPSCs versus WT_Δ iPSCs, which is consistent with increased mitochondrial stress in *NDUFA4*^*−/−*^ iPSCs, non-risk (*T/T*; *T/T*) iPSCs, and *NDUFA4*^Δ^ iPSCs under both mock and infection conditions (Mock and ZIKV^PR^ infection: Figures 6E and 6F; DENV and ZIKV^U^ infection: Figure S6C). Consistently, there was increased mitochondrial stress in iPSC #19 compared to iPSC #1 (Mock and ZIKV^PR^ infection: Figures 6E and 6F; DENV and ZIKV^U^ infection: Figure S6C). To further confirm the mitochondrial stress and the leakage of mtDNA into the cytoplasm, we isolated cytosolic DNA and measured the abundance of mtDNA genes using qPCR experiments (West et al., 2011, 2015; Zhang et al., 2010). The increased leakage of mtDNA genes was observed in *NDUFA4*^*−/−*^ iPSCs versus WT iPSCs, iPSC lines carrying non-risk (*T/T*; *T/T*) versus risk (*G/G*; *C/C*) alleles, *NDUFA4*^Δ^ iPSCs versus WT_Δ iPSCs, and iPSC #19 versus iPSC #1 lines under virus infection conditions (iPSCs in ZIKV^PR^ condition: Figure 6G; iPSCs in DENV and ZIKV^U^ conditions: Figure S6D). Additionally, we performed seahorse assays and found a decreased oxygen consumption rate in *NDUFA4*^*−/−*^ iPSCs versus WT iPSCs, iPSC lines carrying non-risk (*T/T*; *T/T*) versus risk (*G/G*; *C/C*) alleles, *NDUFA4*^Δ^ iPSCs versus WT_Δ iPSCs, and iPSC #19 versus iPSC #1 lines (Figure S6E).

### Loss or reduction of NDUFA4 leads to upregulation of type I interferon signaling

Previous studies showed that mtDNA leakage can trigger type I interferon signaling (West et al., 2011, 2015; Zhang et al., 2010). RNA-sequencing results confirmed enhanced upregulation of the interferon pathway comparing *NDUFA4*^*−/−*^ iPSCs versus WT iPSCs, iPSC lines carrying non-risk (*T/T*; *T/T*) versus risk (*G/G*; *C/C*) alleles, *NDUFA4*^Δ^ iPSCs compared to WT_Δ iPSCs, and iPSC #19 versus iPSC #1 lines (Figure 7A). We also found increased expression levels of interferon-stimulated genes (ISGs), including *ISG15* and *IRF7*, in *NDUFA4*^*−/−*^ iPSCs versus WT iPSCs, iPSC lines carrying non-risk (*T/T*; *T/T*) versus risk (*G/G*; *C/C*) alleles, *NDUFA4*^Δ^ iPSCs versus WT_Δ iPSCs, and iPSC #19 versus iPSC #1 lines under mock or virus-infected conditions (iPSCs in ZIKV^PR^ conditions: Figure 7B; iPSCs in DENV conditions: Figure S7A; iPSCs in ZIKV^U^ conditions: Figure S7B; iPSC-derived airway organoids in SARS-CoV-2 conditions: Figure S7C). To further confirm the role of NDUFA4 in the regulation of virus infection, we treated the low-permissive and permissive iPSC lines with mitochondrial complex IV inhibitor KCN (Hargreaves et al., 2007; Kilbride et al., 2011; Leavesley et al., 2008), and decreased percentages of ZIKV-E+ cells were seen in both permissive and low-permissive iPSC lines (ZIKV^PR^ conditions: Figures 7C and 7D). To further confirm that NDUFA4 inhibits virus infection through activating IFN signaling, we also treated the low-permissive and permissive iPSC lines with a blocking antibody for the type I IFN receptor. Consistent with the hypothesis, increased percentages of ZIKV-E+ cells were seen in both permissive and low-permissive iPSC lines (ZIKV^PR^ conditions: Figures 7E and 7F). Finally, we also found a modest decrease in *NDUFA4* expression level in low-permissive iPSC lines infected with ZIKV, which indicates that cells activate IFN signaling to further decrease viral infection (Figure S7D).

Together, these comparisons suggest that low levels or loss of *NDUFA4* causes basal mitochondrial stress, which leads to the leaking of mtDNA and upregulation of type I interferon signaling. Thus, cells with relatively low levels of NDUFA4 may be primed for elevated interferon signaling, which results in protection during infection through repressed viral replication.

## Discussion

The requirement of a large sample size is a major limiting factor for GWAS, mainly because every individual has been exposed to a different environment, diet, and lifestyle. Compared with population-based GWAS, a tightly controlled cell-culture-based platform can minimize the variation of environmental exposure history, which makes it possible to perform GWAS using a relatively small sample size. Cell-based GWAS using transformed lymphoblastoid cell lines have been performed to associate genetic variation with immune cell levels (Orru et al., 2013), immune cell frequency and differentiation (Roederer et al., 2015), and cytokine response (Mikacenic et al., 2013). However, the genome instability of Epstein-Barr-virus-transformed lymphoblastoid cell lines might overshadow more subtle genetic variation among individuals. Here, we developed an hiPSC-based GWAS. Compared with transformed lymphoblastoid cell lines, the genomes of iPSC lines are more stable and thereby more faithfully reflect the genetic information of individuals. In addition, iPSCs are capable of differentiating into disease-relevant cell types for GWAS. Finally, the CRISPR-based gene editing approach can be broadly used to knock out genes/regions or knock in SNPs, which provides a high-throughput platform to functionally validate the variants or loci identified from GWAS in disease-relevant cell types.

Here, we screened 77 hiPSC lines for relative permissiveness to ZIKV and identified *NDUFA4*, a locus previously unknown to be associated with susceptibility to ZIKV infection. To show the effect is specific to NDUFA4, *NDUFA4* knockout clones were generated from iPSC #9, which has a different genetic background compared to low-permissive cell lines iPSC #15, iPSC #17, and iPSC #19. To avoid the impact of other genetic factors on virus infection, we created isogenic iPSC pairs to study the precise role of *NDUFA4*, SNPs (rs917172 and rs12386620), and a *cis*-regulatory region in ZIKV infection, including *NDUFA4*^*−/−*^ iPSCs versus WT iPSCs, iPSC lines carrying non-risk (*T/T*; *T/T*) versus risk (*G/G*; *C/C*) alleles, and *NDUFA4*^Δ^ iPSCs versus WT_Δ iPSCs. Loss of NDUFA4 led to decreased susceptibility to ZIKV infection. In addition, the genetic variants associated with higher viral infection rates are located in a putative distal *cis*-regulatory region of *NDUFA4*, the ablation of which decreases NDUFA4 expression and decreases sensitivity to viral infection. Moreover, we found that mechanistically, loss or reduction of NDUFA4 induces mitochondrial stress, which leads to the leakage of mtDNA that can be recognized by the innate immune system (Moretton et al., 2017). This leads to the upregulation of type I interferon signaling to block virus infection. As shown in previous studies, type I interferon blocks ZIKV infection of human and mouse brain cells in a dose-dependent manner (van den Pol et al., 2017). A mild increase of type I interferon will not completely block ZIKV infection. Thus, we would not expect that NDUFA4 deletion would completely block virus infection.

In addition to ZIKV, we found that the loss/reduction of NDUFA4 causes decreased SARS-CoV-2 and DENV infection. Transcriptional analysis of lung tissue from healthy donors and COVID-19 patients of an in-house database found that the expression of NDUFA4 is higher in lung tissues from COVID-19 patients compared to healthy donors. The analysis of a dataset with a large patient population with classification of mild, moderate, or severe COVID-19 symptoms found a trend toward higher levels of NDUFA4 expression in severe compared to mild COVID-19 patients. NDUFA4 was also identified in metatranscriptomic sequencing to profile immune signatures in the bronchoalveolar lavage fluid of COVID-19 patients (Zhou et al., 2020). A recent transcriptome-wide association study of 35 clinical infectious disease traits in a cohort of 23,294 individuals also identified NDUFA4 as an infectious-disease-associated gene (Hale et al., 2020). We analyzed the GWAS data from the COVID-19 Host Genetics Initiative (COVID-19 HGI) (https://www.covid19hg.org/) (Niemi et al., 2021) and identified four SNPs associated with NDUFA4 genes with heterogeneity p value less than 0.001 across studies. The COVID-19 HGI database only used computed PC loadings and allele frequencies for the 117,221 SNPs (Niemi et al., 2021). Unfortunately, the two SNPs that we explored in this study, rs917172 and rs12386620, are not included in the 117,221 SNPs of COVID-19 HGI, as often happens when different SNP arrays are used. However, the four SNPs identified in COVID-19 HGI have highlighted a likely contributing role of NUDFA4 in SARS-CoV-2 infection.

In summary, this study provides proof-of-principle for performing GWAS using hiPSC arrays. This tightly controlled platform provides a rapid and cost-efficient system using a relatively small sample size to systematically evaluate association of genetic variants with certain diseases, a paradigm shift for population-based GWAS.

### Limitations of the study

The iPSC lines used in this study were originally derived using Sendai virus. Previous studies have suggested that the Sendai virus vector remains in the cytoplasm of infected cells for a few passages but is diluted quickly and lost completely by passage 10 (Rao and Malik, 2012). Although we do not expect that the iPSC lines still carry Sendai virus after 25 passages, we cannot fully exclude the possible impact of Sendai virus on the permissiveness to ZIKV infection. Also, we defined arbitrarily the permissiveness for infection to facilitate the GWAS in iPSC models. The permissiveness is therefore limited to these iPSC and derivative organoid models at defined viral infection conditions in the laboratory, which cannot be directly generalized to the human population. Using this iPSC GWAS, we identified NDUFA4, associated SNPs, and regulatory regions that contribute to viral infection. However, as with all cell models, the iPSC models will not fully recapitulate the response of human beings in society. There are many additional factors, such as age, health status, exposure risk, and additional genetic factors, that will contribute to the variation of viral susceptibility in humans. In this study, we chose an infection condition that enabled an assay to measure maximal difference between different iPSC lines.

## STAR★Methods

### Key resources table

**Table:** 

REAGENT or RESOURCE	SOURCE	IDENTIFIER
**Antibodies**		

Anti-DNA	Millipore	Cat #CBL186; RRID: AB_93367
Anti-HSP60	Novus	Cat #NBP1-77397; RRID: AB_11004596
Anti-Flavivirus group antigen [D1-4G2-4-15 (4G2)]	GeneTex	Cat #GTX57154; RRID: AB_2887950
Anti-Flavivirus group antigen [D1-4G2-4-15 (4G2)]	Millipore	Cat #MAB10216-I; RRID: AB_827205
Anti-DENV NS3 protein	GeneTex	Cat #GTX124252; RRID: AB_11171668
Anti-human NANOG	R & D	Cat #AF1997; RRID: AB_355097
Anti-OCT-3/4 Antibody	Santa Cruz	Cat #sc-5279; RRID: AB_628051
Anti-SOX2 (D6D9)	Cell Signaling	Cat #3579; RRID: AB_2195767
TRA-1-60 Monoclonal Antibody	BD Biosciences	Cat #560071; RRID: AB_1645604
TRA-1-81 (Podocalyxin) Monoclonal Antibody	Thermo Scientific	Cat #14-8883-82; RRID: AB_891614
Anti-FOXJ1	Thermo Fisher Scientific	Cat #14-9965-82; RRID: AB_1548835
Anti-Acetyl-α-Tubulin (Lys40) (D20G3)	Cell Signaling	Cat #5335S; RRID: AB_10544694
Anti-MAP2 antibody	Abcam	Cat #ab5392; RRID: AB_2138153
Anti-E-Cadherin (4A2)	Cell Signaling	Cat #14472S; RRID: AB_2728770
Anti-SARS-CoV/SARS-CoV-2 Nucleocapsid	SinoBiological	Cat #40143-R001; RRID: AB_2827974
Anti-NDUFA4 antibody	Abcam	Cat #ab129752; RRID: AB_11155881
Anti-β actin	Thermo Fisher Scientific	Cat # MA1-140; RRID: AB_2536844
Anti-STEM121 human antigen	Takara	Cat #Y40410; RRID: AB_2801314
Anti-Mucin 5AC antibody	Abcam	Cat #ab3649; RRID: AB_2146844
Anti- IFN-alpha/beta R1	R & D	Cat #AF245; RRID: AB_355270
Anti- IFN-alpha/beta R2	R & D	Cat #MAB4015; RRID: AB_2122619
Donkey anti-Mouse IgG (H + L) Highly Cross-Adsorbed Secondary Antibody, Alexa Fluor 488	Thermo Fisher Scientific	Cat #A-21202; RRID: AB_141607
Donkey anti-Mouse IgG (H + L) Highly Cross-Adsorbed Secondary Antibody, Alexa Fluor 594	Thermo Fisher Scientific	Cat #A-21203; RRID: AB_141633
Donkey anti-Rabbit IgG (H + L) Secondary Antibody, Alexa Fluor 594 conjugate	Thermo Fisher Scientific	Cat #A-21207; RRID: AB_141637
Donkey anti-Rabbit IgG (H + L) Secondary Antibody, Alexa Fluor 647 conjugate	Thermo Fisher Scientific	Cat #A-31573; RRID: AB_2536183
Donkey anti-Mouse IgG (H + L) Secondary Antibody, Alexa Fluor 647	Thermo Fisher Scientific	Cat #A-31571; RRID: AB_162542
680RD Donkey anti-Mouse IgG Secondary Antibody	Li-Cor	Cat #926–68072; RRID: AB_10953628
800CW Donkey anti-Rabbit IgG Secondary Antibody	Li-Cor	Cat #926–32213; RRID: AB_621848
DAPI	Thermo Fisher Scientific	Cat# D1306; RRID:AB_2629482

**Bacterial and virus strains**		

Zika Virus (Strain: MR 766)	ZeptoMetrix	Cat# 0810521CF
Zika Virus (Strain: PRVABC59)	ZeptoMetrix	Cat# 0810525CF
SARS-CoV-2 Isolate USA-WA1/2020	BEI Resources	Cat# NR-52281
Dengue virus type 2	ATCC	Cat# VR-1584

**Biological samples**		

Patient samples	This paper	N/A

**Chemicals, peptides, and recombinant proteins**		

Y-27632	MedchemExpress	#HY-10583
CHIR99021	Cayman Chemical	#13122
Retinoic acid	Sigma Aldrich	#R2625-500MG
L-Ascorbic acid	Sigma Aldrich	#A4544-100G
SB431542	R&D Systems	#1614/50
Dorsomorphin dihydrochloride	R&D Systems	#3093/50
Dexamethasone	Sigma-Aldrich	#D4902
Potassium cyanide	Sigma-Aldrich	#60178
8-Bromo-cAMP	Sigma-Aldrich	#B7880
IBMX	Sigma-Aldrich	#I5879
DAPT	R&D Systems	#2634
Activin A	R&D Systems	#338-AC-500/CF
Recombinant Human bFGF Protein	Peprotech	#100-18B-500UG
Recombinant Human BMP-4 Protein	R & D Systems	#314-BP
Recombinant Human FGF-10 Protein	R&D Systems	#345-FG-250

**Critical commercial assays**		

TruSeq Stranded mRNA LP	Illumina	#20020594
Seahorse XF Cell Mito Stress Test Kit	AGILENT TECHNOLOGIES	#103015-100

**Deposited data**		

RNA-seq	This Paper	GEO: GSE182376, GSE203401

**Experimental models: Cell lines**		

293T	ATCC	#CRL-11268
Vero E6	ATCC	#CRL-1586
iPSC cell lines	Paull et al., 2015	N/A

**Software and algorithms**		

Rstudio	Rstudio	https://rstudio.com
Seurat R package v3.1.4	Butler et al., 2018	https://satijalab.org/seurat/
DAVID6.8	LHRI	https://david.ncifcrf.gov/home.jsp
Adobe illustrator CC2017	Adobe	https://www.adobe.com/product/photoshop.html
Graphpad Prism 9	Graphpad software	https://www.graphpad.com

### Resource availability

#### Lead contact

Further information and requests for resources and reagents should be directed to and will be fulfilled by the Lead Contact, Shuibing Chen (shc2034@med.cornell.edu).

#### Materials availability

*NDUFA4*^*−/−*^, WT iPSCs, iPSCs carrying risk (*G/G*; *C/C*) or non-risk (*T/T*; *T/T*) alleles, and *NDUFA4*^Δ^, WT_Δ iPSCs are available upon request under appropriate Material Transfer Agreement.

### Experimental model and subject details

#### Human samples

Tissue was acquired from surgical waste (vas deferens segments) of vasectomies provided by FTLN and GJW. All available samples were used in this study. The tissue procurement operates under Institutional Review Board (IRB) approved protocol and follows guidelines set by HIPAA. Experiments using samples from human subjects were conducted in accordance with local regulations and with the approval of the institutional review board at the Instituto de Medicina Integral Professor Fernando Figueira under protocol number 79872317.1.0000.5201 and approval number 2.518.543.

#### Viral strains

##### Flaviviruses

ZIKV^PR^ (PRVABC59) strain and ZIKV^U^ (MR766) strain were obtained from ZeptoMetrix and titered by plaque assay using Vero cells.

Dengue virus stocks were obtained from ATCC (VR-1584). Titers were determined by plaque assay using Vero cells.

##### SARS-CoV-2 virus

SARS-CoV-2 isolate USA-WA1/2020 (NR-52281) was deposited by the Center for Disease Control and Prevention and obtained through BEI Resources, NIAID, NIH. SARS-CoV-2 was propagated in Vero E6 cells in DMEM supplemented with 2% FBS as described previously (Yang et al., 2020). Virus stocks were filtered and concentrated by centrifugation using Amicon Ultra-15 Centrifugal filter units as previously described (Nilsson-Payant et al., 2021). Infectious titers were determined by plaque assays in Vero E6 cells in Dulbecco’s Minimum Essential Media supplemented with 2% FBS, 1% pen/strep and 0.8% Sea-Plaque agarose as has been described previously (Blanco-Melo et al., 2020; Samelson et al., 2022). All work involving SARS-CoV-2 was performed in the CDC/USDA-approved BSL-3 facility of the Global Health and Emerging Pathogens Institute at the Icahn School of Medicine at Mount Sinai or in the CDC/USDA-approved BSL-3 facility of the Vilcek Institute of Graduate Biomedical Sciences at NYU Langone Health in accordance with institutional biosafety requirements.

#### Cell lines

hiPSCs were grown on matrigel-coated plates with mTeSR1 medium (Stem Cell Technology). Cells were maintained at 37 °C with 5% CO_2_. The hiPSC library was obtained from the New York Stem Cell Foundation (Paull et al., 2015). The gender and ethnicity information were provided in Table S1. Human SNP Array 6.0 was used for the authentication of hiPSC lines. Both male and female iPSC lines were included and reported in this study.

Vero E6 (Female, African green monkey [*Chlorocebus aethiops*] kidney) were cultured in Dulbecco’s Modified Eagle Medium (DMEM) supplemented with 10% fetal bovine serum (FBS) and 100 U/mL penicillin and 100 μg/mL streptomycin, and maintained at 37°C with 5% CO_2_.

HEK 293T cells (Female) were grown in DMEM media supplemented with 10% FBS, 1% PenStrep (Gibco, Grand Island, NY) at 37°C in 5% CO_2_ atmosphere.

### Method details

#### hiPSC culture and screening

iPSC lines derived from 77 individuals were split onto 96-well plates at 10,000 cells/well, with 3 repeats for each line. The iPSC lines were of similar density (∼50% density) two days after splitting. iPSCs were infected with ZIKV (ZIKV^U^, MOI = 0.25; ZIKV^PR^ strain MOI = 1) for 2 h and changed to virus-free medium. At 72 hpi, cells were fixed, stained with the antibody against the ZIKV envelope protein (ZIKV-E), and analyzed with the ImageXpress Micro Widefield High-Content Analysis System to calculate the percentage of ZIKV-E^+^ cells for each hiPSC line.

#### Virus infections

*ZIKV infection:* iPSCs or iPSC-derived cells were plated one day before infection and then infected with ZIKV. The MOI used in each experiment is indicated in the main text and/or figure legends. Cells were incubated with medium containing ZIKV at 37°C for 2 h. After infection, fresh medium was replaced and cells were maintained at 37°C for 48 or 72 h as indicated.

*DENV infection:* iPSCs were plated 2 days prior to infection and grown at 37°C. The cells were infected with DENV at an MOI of 1 by incubating them with virus at 37°C for 2 h. After infection, the virus inoculum was removed, the cells were washed with 1X PBS, and fresh medium was added. The cells were harvested after 72 h of incubation at 37°C.

*SARS-CoV-2 infection:* Airway organoids were dissociated into small clusters and replated into 24 or 96-well plates for 24 h. Then, airway organoids were infected with SARS-CoV-2 virus at an MOI of 0.1 and incubated at 37°C for 24 h. Supernatants containing infectious virus were collected and stored at −80C for plaque assays. Infected cells were either lysed in TRIzol for RNA analysis or fixed in 5% formaldehyde for 24 h for immunofluorescence staining.

#### SNP array and GWAS analysis

High-throughput genotyping of 906,600 SNPs was performed on Affymetrix Genome-Wide Human SNP Array 6.0 (Santa Clara, California, USA) at the core facility of Albert Einstein College of Medicine following the manufacturer’s instructions (Affymetrix, Inc., Santa Clara, California, USA). Genotyping was performed using the default parameters in the Birdseed v2 algorithm of Genotyping Console (GTC) 4.2 software (Affymetrix). As a quality control for the genotyping, contrast QC values were calculated as implemented in GTC 4.2, and samples used passed the recommended values. Genome annotations applied in data analysis refer to the human reference assembly GRCh37/hg19 as provided by the Affymetrix annotation file. In this study, SNPs with low minor allele frequencies (<0.05), low call rates (<95%), and inconsistent genotype frequencies with Hardy–Weinberg equilibrium (p < 1.0 × 10^−5^) were excluded. In addition, allosome SNPs were not analyzed. After quality control, a total of 691,822 common SNPs were included in the current analysis. An iterative procedure was used to simultaneously estimate principal components reflecting population structure. Logistic regression was applied to examine associations of SNPs with sensitivity of ZIKV infection (low permissive lines vs. permissive lines) under an additive genetic model adjusted for sex and two principal components. Analyses were performed using R (version 3.3.2 R Foundation).

#### Generation of knockout or knock-in hiPSC lines

CRISPR sgRNA sequences were designed using https://www.benchling.com/crispr/. The target sequences are listed in Table S4. The sgRNA was cloned into the pX330-U6-Chimeric_BB-CBh-hSpCas9 vector (Addgene plasmid #42230). To knockout the target gene or knock-in the SNP allele, iPSC #9 was dissociated using Accutase (Stem Cell Technology) and electroporated (1x10^6^ cells per sample) with 4 μg sgRNA-construct plasmids using Human Stem Cell NucleofectorTM solution (Lonza) following manufacturer’s instructions. Cells were seeded in 2 wells of 24-well plates. 4 days later, hiPSCs were dissociated into single cells with Accutase and subcloned. 10 μM Y-27632 was used to improve the survival of single colonies. 10 days later, individual colonies derived from single cells were picked, mechanically disaggregated and replated into two individual wells of 96-well plates. A portion of the cells was analyzed by DNA sequencing. For biallelic frameshift mutants, we chose either homozygous mutants or compound heterozygous mutants. Considering the potential nonspecific effects associated with the gene targeting process, wildtype clonal lines from the same targeting experiments were always included as controls. To knockout regulatory region, a pair of sgRNA plasmids were generated and electroporated to iPSC #9. After sub-cloning, the region knockout clones were screened by PCR.

To knock in risk alleles of SNPs, a sgRNA target was cloned into the pX330-U6-Chimeric_BB-CBh-hSpCas9 vector, was co-electroporated with a donor template ssODN to the iPSC-9 (sgRNA targets and ssODN sequences were listed in below). Single-cell clones were isolated and evaluated by Sanger sequencing to screen the SNP lines.

#### Construction of NDUFA4 overexpression cell lines

The control overexpression plasmid and NDUFA4 overexpression plasmid were obtained from VectorBuilder (VB900004-0599xvh). The overexpression plasmids were transfected into 293T cells together with pMD2G and psPAX2 plasmids using calcium phosphate transfection. Lentivirus was collected and concentrated using Lenti-X Concentrator (Clontech) at 48 and 72 hpi. Then, iPSC lines #15, #19 and *NDUFA4*^−/−^ cells were infected with lentivirus for 48 h and sorted based on GFP expression.

#### Differentiation of cerebral organoids

Cerebral organoids were differentiated using STEMdiff Cerebral Organoid Kit from Stem Cell Technologies (#08570) following manufacturer’s instructions. At day 20, age-matched cerebral organoids were infected with ZIKV^PR^ (3 × 10^6^ PFU/mL) or ZIKV^U^ (5 × 10^5^ PFU/mL) overnight. At 72 hpi, organoids were fixed in 4%PFA for two days and then transferred to 30% sucrose, followed by snap-freezing in O.C.T (Fisher Scientific).

#### Differentiation of airway organoids

Airway organoids were differentiated using a previously reported protocol (Jacob et al., 2017). Briefly, hiPSCs were dissociated into single cells using Gentle Cell Dissociation Reagent (Stem Cell Technologies, #07174) and 2x10^6^ cells were plated to 1 well of matrigel-coated 6-well plates. 24 h later, medium was changed to RPMI-1640 supplemented with 1×Glutamax (Thermo Fisher Scientific), 50 μg/mL Normocin, 100 ng/mL Activin A (R&D systems), and 2 μM of CHIR99021 (Cayman Chemical) for 24 h. The medium was then changed to RPMI-1640 medium supplemented with 1×Glutamax (Thermo Fisher Scientific), 50 μg/mL Normocin, 0.2% fetal bovine serum (FBS, Corning), 100 ng/mL Activin A (R&D systems) for 2 days. Then, cells were dissociated again using Gentle Cell Dissociation Reagent (Stem Cell Technologies, #07174) and passaged at ratio 1:3 or 1:4 into matrigel-coated plates in DS/SB medium supplemented with 10 μM Y-27632. 24 h later, medium was switched to DS/SB medium without Y-27632. After 72 h’ induction, cells were changed to CBRa medium, which was refreshed every 48 h. 3–5 days’ later, CD47^hi^CD26^lo^ lung progenitor cells were sorted and resuspended at 1000 cells/μL in undiluted matrigel matrix, which was replated in 50 μL drops into 1 well of 24-well plates. Cells began to form epithelial organoids after several days of culture and can be passaged until the drop is filled with cells. DS/SB medium and CBRa medium was prepared according to the published protocol (Jacob et al., 2017).

#### qRT-PCR

Total RNA samples were prepared from cells with RNeasy Plus Mini Kit (Qiagen, #74136) and reverse transcribed (RT) with high-capacity cDNA reverse transcription Kit supplemented with RNase inhibitor (Thermo Fisher, # 4,374,966). For the quantification of (+) and (−) strands of ZIKV vRNAs, strand specific primers were used in RT. Human β-Actin was employed as an internal reference. PrimeTime Gene Expression 2X Master Mix (IDT, #1055772) and probes for ZIKV vRNA and human β-Actin were used to perform qPCR reactions. For quantification of genes, random primers were used in RT. Human β-Actin was employed as an internal reference. The sequences of primers/probes are provided in Table S6.

#### Immunohistochemistry

Cells were fixed in 4% PFA for 20 min at room temperature, blocked in Mg^2+^/Ca^2+^ free PBS plus 5% horse serum and 0.3% Triton X- for 1 h at room temperature, and then incubated with primary antibody at 4°C overnight. The information for primary antibodies is provided in Table S7. Secondary antibodies included donkey anti-mouse, goat, rabbit or chicken antibodies conjugated with Alexa Fluor 488, Alexa Fluor 594 or Alexa Fluor 647 fluorophores (1:500, Life Technologies). Nuclei were counterstained by DAPI. Images were acquired using an LSM 880 Laser Scanning Confocal Microscope and processed with Zen software. Quantification was performed using ImageJ (NIH) software.

#### Vero cell assay

Vero cells were maintained in DMEM medium plus 10% fetal bovine serum. Cells were plated into 96-well plates at a density of 25,000 cells/well. After overnight incubation, supernatants were collected from ZIKV infected cell cultures and then diluted serially from 10- to 10^8^-fold to infect Vero cells in 96-well plates for 2 h. After infection, medium was replaced with semi-solid medium of alpha-MEM containing 10% fetal bovine serum and 1% methylcellulose. Two days after infection, cells were fixed with 4% PFA and stained with anti-ZIKV E.

#### Western blotting

Cells were collected in Pierce RIPA buffer (Thermo Fisher Scientific) plus HALT protease inhibitor cocktail (1:100) (Thermo Fisher Scientific) and lysates loaded on 12% NuPage Bis-Tris pre-cast gels (Thermo Fisher Scientific). After separation by electrophoresis, proteins were transferred to 0.2 μm nitrocellulose membranes (Thermo Fisher Scientific). Membranes were blocked with 5% milk in TBS +0.1% Tween and incubated with primary antibody overnight. Information for primary antibodies is provided in Table S7. Membranes were washed and incubated with secondary antibody for 1 h at room temperature in 5% milk-TBS-0.1% Tween and developed using Super-Signal West Pico PLUS chemiluminescent substrate (Thermo Fisher Scientific). Human β-Actin was employed as an internal reference.

#### Viral entry assay

ZIKV reporter virus particles (RVP) were generated as previously described (Robbiani et al., 2017). For the RVP entry assay, WT, *NDUFA4*^*−/−*^ and *NDUFA4*^*ΔSNP*^ iPSCs were seeded in 24-well plates at a density of 62,500 cells per well, and two days later, infected with ZIKV RVP. As the West Nile virus (WNV) mini-genome packaged inside the ZIKV RVP is replication-competent, the luciferase signal generated in infected cells is the cumulative outcome of translation of the incoming RNA as well as newly generated, replication-dependent, RNA. To offset the effect of WNV replication and specifically monitor virus entry, a flavivirus replication inhibitor, NITD008, was added to the culture medium 24 h before infection and maintained in the medium during and after infection. RVP-infected cells were harvested at 24 hpi, and luciferase activity measured using FLUOstar Omega (BMD Labtech, Germany).

#### Virus replicon assay

ZIKV replicons have been previously reported (Rusanov et al., 2018). To determine whether NDUFA4 is involved in flavivirus genome replication, WT, *NDUFA4*^*−/−*^ and *NDUFA4*^*ΔSNP*^ iPSCs were transfected with the *in vitro* transcribed ZIKV replicon RNA. iPSCs were plated into 24-well plates at 62,500 cells per well. Transfection mixes for each well were prepared by mixing 0.1 μg of the replicon RNA with 0.2 μL each of the mRNA boost reagent and TransIT-mRNA reagent (TransIT-mRNA transfection kit; Mirus, WI). At 24 hpi, cells were lysed in 1X cell culture lysis reagent (Promega, WI) followed by measurement of the Renilla luciferase activity.

#### Luciferase reporter assays of the SNPs associated with NDUFA4

DNA sequencing ranging from 140 bp to 249 bp around the identified SNPs associated with *NDUFA4* were amplified using forward and reverse primers adding restriction sites- Kpn I and Nhe I (New England Biolabs, Ipswich, MA) for cloning into corresponding sites of pGL4.10 (luc2) luciferase reporter vector (Promega, Madison, WI) with restriction enzyme digestion and T4 ligase ligation method. Constructed luciferase reporter vectors were characterized by restriction analysis and DNA sequencing. Site directed mutation for each SNP was performed with Q5 Site-Directed Mutagenesis Kit (Promega, Madison, WI) according to manufacture protocol and the primers used for site directed mutagenesis were designed with the NEBaseChanger (Promega, Madison, WI).

293T cells were seeded in 24-well plates, grown to 80–90% confluence, and transfected by PEI (Sigma, St. Louis, MO) according to the manufacturer’s instructions. Cells were transfected with either the empty pGL4.10 (luc2) luciferase reporter vectors or vectors containing SNPs or their mutations and Renilla luciferase control reporter vector (Promega, Madison, WI) for transfection efficiency normalization. After 48 h of transfection, cells were harvested in passive lysis buffer (Promega, Madison, WI). Luciferase activity was measured using the Dual-Luciferase Reporter Assay system kit (Promega, Madison, WI) according to manufacture procedure on a Synergy H1 microplate reader (Winooski, VT).

#### RNA-sequencing and analysis

Cells were lysed to prepare total RNA for sequencing using the RNeasy plus mini kit (Qiagen, #74136). cDNA libraries were prepared with the TruSeq RNA Sample Preparation kit (Illumina), and sequenced with the HiSeq4000 sequencer (Illumina) at the Weill Cornell Genomics Resources Core Facility. Sequencing reads were cleaned by trimming adapter sequences and low quality bases using cutadapt (https://cutadapt.readthedocs.io/en/stable/), and aligned to human genome (GRCh38) using HISAT2 (version = 2.1.0) (Kim et al., 2019), and the raw counts were extracted from bam files by featureCounts function (from package subread version = 1.6.4) (Liao et al., 2019). Cufflinks was used to measure transcript abundance in Fragments Per Kilobase of exon model per Million mapped reads (FPKM). Heatmap plots were generated using the “Heatmapper”. Gene Ontology analysis was carried out using GSEA software (4.0.3).

#### Detection of mtDNA in cytosolic extracts

Digitonin extracts from iPSCs were generated as described previously (West et al., 2015). Briefly, cells were each divided into two equal aliquots, and one aliquot was resuspended in 500 μL of 50 mM NaOH and boiled for 30 min to solubilize DNA. 1 M TrisHCl pH 8 was added to neutralize the pH, and these extracts served as normalization controls for total mtDNA. The second equal part was resuspended in roughly 500 μL buffer containing 150 mM NaCl, 50 mM HEPES pH 7.4, and 15–25 mg/mL digitonin. The homogenates were incubated end over end for 10 min to allow selective plasma membrane permeabilization, then centrifuged at 1000g for 3 min three times to pellet intact cells. The cytosolic supernatants were transferred to new tubes and spun at 17000g for 10 min to yield cytosolic preparations free of nuclear, mitochondrial and endoplasmic recticulum contamination. DNA was then isolated from these pure cytosolic fractions using QIAQuick Nucleotide Removal Columns (QIAGEN). qPCR was performed on both whole-cell extracts and cytosolic fractions using nuclear DNA primers (Tert) and mtDNA primers (D loop1-3, Cytb, 16S and Nd4), and the CT values obtained for mtDNA abundance for whole-cell extracts served as normalization controls for the mtDNA values obtained from the cytosolic fractions.

#### Seahorse analysis

Seahorse assays were performed using the Agilent Seahorse XF Cell Mito Stress Test Kit (Agilent Technologies) to measure the oxygen consumption rate (OCR) of cells on the Seahorse XFe 96 Flux Analyzer. Briefly, 4x10^4^ hiPSCs were plated in the 96 well Seahorse XF Cell Culture Microplate and the sensor cartridge was hydrated in Seahorse XF Calibrant at 37°C in a non-CO_2_ incubator overnight. The day of assay, cells were washed three times using assay medium (Seahorse XF DMEM medium supplemented with 1 mM pyruvate, 2 mM glutamine and 10 mM glucose). 1.5 μM oligomycin, 1.0 μM FCCP and 0.5 μM rotenone/antimycin A were used to test the oxygen consumption rate.

### Quantification and statistical analysis

Data are shown as mean ± SD For a two sample-group comparison, unpaired two-tailed Student’s *t* test was used to test the statistical significance of the data. For low permissive and permissive cell line comparisons, we averaged the 3 technical replicates within each cell line, then used the averages for an unpaired two-tailed Student’s *t* test. For two phenotypes and two colonies comparisons, we used two-way ANOVA analysis followed by Tukey post-hoc test with multiple testing correction. For low-permissive and permissive cell lines’ replication curves, we averaged the technical replicates for each time point within each cell line and used the averages of each cell line for an unpaired two-tailed Student’s *t* test for each time point. For other replication curves, we used two-way ANOVA analysis followed by Tukey post-hoc test with multiple testing correction for each time point. N = 3 independent biological replicates were used for all experiments unless otherwise indicated. n.s. indicates a non-significant difference. ^∗^p < 0.05, ^∗∗^p < 0.01 and ^∗∗∗^p < 0.001. For statistical analyses, we used GraphPad Prism 8.4.1 software.

## Data Availability

RNA-seq data have been deposited at GEO and are publicly available as of the date of publication. Accession numbers are listed in the key resources table. All original code has been deposited at https://github.com/shuibingchen/iPSC-array-and-ZIKV and is publicly available as of the date of publication. Any additional information required to reanalyze the data reported in this paper is available from the lead contact upon request.

## Supplemental information

Document S1. Figures S1–S7 and Tables S1 and S3–S7

Table S2. Information of top 100 SNPs identified from GWAS, related to Figure 1

Document S2. Article plus supplemental information

## References

[bib1] Abdulrahman A., Mallah S.I., Alqahtani M. (2021). COVID-19 viral load not associated with disease severity: findings from a retrospective cohort study. BMC Infect. Dis..

[bib2] Balsa E., Marco R., Perales-Clemente E., Szklarczyk R., Calvo E., Landázuri M., Enríquez J. (2012). NDUFA4 is a subunit of complex IV of the mammalian electron transport chain. Cell Metab.

[bib4] Blanco-Melo D., Nilsson-Payant B.E., Liu W.C., Uhl S., Hoagland D., Moller R., Jordan T.X., Oishi K., Panis M., Sachs D. (2020). Imbalanced Host Response to SARS-CoV-2 Drives Development of COVID-19. Cell.

[bib5] Duan X., Tang X., Nair M.S., Zhang T., Qiu Y., Zhang W., Wang P., Huang Y., Xiang J., Wang H. (2021). An airway organoid-based screen identifies a role for the HIF1α-glycolysis axis in SARS-CoV-2 infection. Cell Rep..

[bib54] Butler A., Hoffman P., Smibert P., Papalexi E., Satija R. (2018). Integrating single-cell transcriptomic data across different conditions, technologies, and species. Nat Biotechnol.

[bib6] Degenhardt F., Bujanda L., Buti M., Albillos A., Invernizzi P., Fernández J., Prati D., Baselli G., Asselta R., The Severe Covid-19 GWAS Group (2020). Genomewide Association Study of Severe Covid-19 with Respiratory Failure. N. Engl. J. Med..

[bib7] Gabriel E., Ramani A., Karow U., Gottardo M., Natarajan K., Gooi L.M., Goranci-Buzhala G., Krut O., Peters F., Nikolic M. (2017). Recent Zika Virus Isolates Induce Premature Differentiation of Neural Progenitors in Human Brain Organoids. Cell Stem Cell.

[bib8] Gibson G. (2010). Hints of hidden heritability in GWAS. Nat. Genet..

[bib9] Hale A.T., Zhou D., Bastarache L., Wang L., Zinkel S.S., Schiff S.J., Ko D.C., Gamazon E.R. (2020). The genetic architecture of human infectious diseases and pathogen-induced cellular phenotypes. medRxiv.

[bib10] Han Y., Duan X., Yang L., Nilsson-Payant B.E., Wang P., Duan F., Tang X., Yaron T.M., Zhang T., Uhl S. (2021). Identification of SARS-CoV-2 inhibitors using lung and colonic organoids. Nature.

[bib11] Hargreaves I.P., Duncan A.J., Wu L., Agrawal A., Land J.M., Heales S. (2007). Inhibition of mitochondrial complex IV leads to secondary loss complex II-III activity: implications for the pathogenesis and treatment of mitochondrial encephalomyopathies. Mitochondrion.

[bib12] Hooper S.D., Johansson A.C., Tellgren-Roth C., Stattin E.L., Dahl N., Cavelier L., Feuk L. (2012). Genome-wide sequencing for the identification of rearrangements associated with Tourette syndrome and obsessive-compulsive disorder. BMC Med. Genet..

[bib13] Jacob A., Morley M., Hawkins F., McCauley K.B., Jean J.C., Heins H., Na C.L., Weaver T.E., Vedaie M., Hurley K. (2017). Differentiation of Human Pluripotent Stem Cells into Functional Lung Alveolar Epithelial Cells. Cell Stem Cell.

[bib14] Kadenbach B. (2017). Regulation of Mammalian 13-Subunit Cytochrome c Oxidase and Binding of other Proteins: Role of NDUFA4. Trends Endocrinol Metab.

[bib15] Kilbride S.M., Gluchowska S.A., Telford J.E., O'Sullivan C., Davey G.P. (2011). High-level inhibition of mitochondrial complexes III and IV is required to increase glutamate release from the nerve terminal. Mol. Neurodegener..

[bib16] Kim D., Paggi J.M., Park C., Bennett C., Salzberg S.L. (2019). Graph-based genome alignment and genotyping with HISAT2 and HISAT-genotype. Nat. Biotechnol..

[bib17] Leavesley H.B., Li L., Prabhakaran K., Borowitz J.L., Isom G.E. (2008). Interaction of cyanide and nitric oxide with cytochrome c oxidase: implications for acute cyanide toxicity. Toxicol. Sci..

[bib18] Liao Y., Smyth G.K., Shi W. (2019). The R package Rsubread is easier, faster, cheaper and better for alignment and quantification of RNA sequencing reads. Nucleic Acids Res..

[bib19] Matusali G., Houzet L., Satie A.P., Mahé D., Aubry F., Couderc T., Frouard J., Bourgeau S., Bensalah K., Lavoué S. (2018). Zika virus infects human testicular tissue and germ cells. J. Clin. Invest..

[bib20] Mikacenic C., Reiner A.P., Holden T.D., Nickerson D.A., Wurfel M.M. (2013). Variation in the TLR10/TLR1/TLR6 locus is the major genetic determinant of interindividual difference in TLR1/2-mediated responses. Genes Immun.

[bib21] Mlakar J., Korva M., Tul N., Popović M., Poljšak-Prijatelj M., Mraz J., Kolenc M., Resman Rus K., Vesnaver Vipotnik T., Fabjan Vodušek V. (2016). Zika Virus Associated with Microcephaly. N. Engl. J. Med..

[bib22] Moretton A., Morel F., Macao B., Lachaume P., Ishak L., Lefebvre M., Garreau-Balandier I., Vernet P., Falkenberg M., Farge G. (2017). Selective mitochondrial DNA degradation following double-strand breaks. PLoS One.

[bib23] Mozzi A., Pontremoli C., Sironi M. (2018). Genetic susceptibility to infectious diseases: Current status and future perspectives from genome-wide approaches. Infect. Genet. Evol..

[bib24] Müller F.E., Braun M., Syring I., Klümper N., Schmidt D., Perner S., Hauser S., Müller S.C., Ellinger J. (2015). NDUFA4 expression in clear cell renal cell carcinoma is predictive for cancer-specific survival. Am J Cancer Res.

[bib25] Niemi M.E.K., Karjalainen J., Liao R.G., Neale B.M., Daly M., Ganna A., Pathak G.A., Andrews S.J., Kanai M., Veerapen K. (2021). Mapping the human genetic architecture of COVID-19. Nature.

[bib26] Nilsson-Payant B.E., Uhl S., Grimont A., Doane A.S., Cohen P., Patel R.S., Higgins C.A., Acklin J.A., Bram Y., Chandar V. (2021). The NF-κB Transcriptional Footprint Is Essential for SARS-CoV-2 Replication. J. Virol..

[bib27] Orru V., Steri M., Sole G., Sidore C., Virdis F., Dei M., Lai S., Zoledziewska M., Busonero F., Mulas A. (2013). Genetic variants regulating immune cell levels in health and disease. Cell.

[bib28] Pairo-Castineira E., Clohisey S., Klaric L., Bretherick A.D., Rawlik K., Pasko D., Walker S., Parkinson N., Fourman M.H., Russell C.D. (2021). Genetic mechanisms of critical illness in COVID-19. Nature.

[bib29] Paull D., Sevilla A., Zhou H., Hahn A.K., Kim H., Napolitano C., Tsankov A., Shang L., Krumholz K., Jagadeesan P. (2015). Automated, high-throughput derivation, characterization and differentiation of induced pluripotent stem cells. Nat. Methods.

[bib30] Pitceathly R., Rahman S., Wedatilake Y., Polke J., Cirak S., Foley A., Sailer A., Hurles M., Stalker J., Hargreaves I. (2013). NDUFA4 mutations underlie dysfunction of a cytochrome c oxidase subunit linked to human neurological disease. Cell Rep..

[bib31] Qian X., Nguyen H., Song M., Hadiono C., Ogden S., Hammack C., Yao B., Hamersky G., Jacob F., Zhong C. (2016). Brain-Region-Specific Organoids Using Mini-bioreactors for Modeling ZIKV Exposure. Cell.

[bib32] Quiros P.M., Prado M.A., Zamboni N., D'Amico D., Williams R.W., Finley D., Gygi S.P., Auwerx J. (2017). Multi-omics analysis identifies ATF4 as a key regulator of the mitochondrial stress response in mammals. J. Cell Biol..

[bib33] Rao M.S., Malik N. (2012). Assessing iPSC reprogramming methods for their suitability in translational medicine. J. Cell. Biochem..

[bib34] Robbiani D.F., Bozzacco L., Keeffe J.R., Khouri R., Olsen P.C., Gazumyan A., Schaefer-Babajew D., Avila-Rios S., Nogueira L., Patel R. (2017). Recurrent Potent Human Neutralizing Antibodies to Zika Virus in Brazil and Mexico. Cell.

[bib35] Robinson C.L., Chong A.C.N., Ashbrook A.W., Jeng G., Jin J., Chen H., Tang E.I., Martin L.A., Kim R.S., Kenyon R.M. (2018). Male germ cells support long-term propagation of Zika virus. Nat. Commun..

[bib36] Roederer M., Quaye L., Mangino M., Beddall M., Mahnke Y., Chattopadhyay P., Tosi I., Napolitano L., Terranova Barberio M., Menni C. (2015). The genetic architecture of the human immune system: a bioresource for autoimmunity and disease pathogenesis. Cell.

[bib37] Rusanov T., Kent T., Saeed M., Hoang T.M., Thomas C., Rice C.M., Pomerantz R.T. (2018). Identification of a Small Interface between the Methyltransferase and RNA Polymerase of NS5 that is Essential for Zika Virus Replication. Sci. Rep..

[bib38] Saeed M., Andreo U., Chung H.Y., Espiritu C., Branch A.D., Silva J.M., Rice C.M. (2015). SEC14L2 enables pan-genotype HCV replication in cell culture. Nature.

[bib39] Salinas R.E., Ogohara C., Thomas M.I., Shukla K.P., Miller S.I., Ko D.C. (2014). A cellular genome-wide association study reveals human variation in microtubule stability and a role in inflammatory cell death. Mol. Biol. Cell.

[bib40] Samelson A.J., Tran Q.D., Robinot R., Carrau L., Rezelj V.V., Kain A.M., Chen M., Ramadoss G.N., Guo X., Lim S.A. (2022). BRD2 inhibition blocks SARS-CoV-2 infection by reducing transcription of the host cell receptor ACE2. Nat. Cell Biol..

[bib41] Tang H., Hammack C., Ogden S., Wen Z., Qian X., Li Y., Yao B., Shin J., Zhang F., Lee E. (2016). Zika Virus Infects Human Cortical Neural Progenitors and Attenuates Their Growth. Cell Stem Cell.

[bib42] van den Pol A.N., Mao G., Yang Y., Ornaghi S., Davis J.N. (2017). Zika Virus Targeting in the Developing Brain. J. Neurosci..

[bib43] Wang L., Pittman K.J., Barker J.R., Salinas R.E., Stanaway I.B., Williams G.D., Carroll R.J., Balmat T., Ingham A., Gopalakrishnan A.M. (2018). An Atlas of Genetic Variation Linking Pathogen-Induced Cellular Traits to Human Disease. Cell Host Microbe.

[bib44] West A.P., Khoury-Hanold W., Staron M., Tal M.C., Pineda C.M., Lang S.M., Bestwick M., Duguay B.A., Raimundo N., MacDuff D.A. (2015). Mitochondrial DNA stress primes the antiviral innate immune response. Nature.

[bib45] West A.P., Shadel G.S., Ghosh S. (2011). Mitochondria in innate immune responses. Nat. Rev. Immunol..

[bib46] Yagil C., Varadi-Levi R., Yagil Y. (2018). A novel mutation in the NADH dehydrogenase (ubiquinone) 1 alpha subcomplex 4 (Ndufa4) gene links mitochondrial dysfunction to the development of diabetes in a rodent model. Dis Model Mech.

[bib47] Yang L., Han Y., Nilsson-Payant B.E., Gupta V., Wang P., Duan X., Tang X., Zhu J., Zhao Z., Jaffré F. (2020). A Human Pluripotent Stem Cell-based Platform to Study SARS-CoV-2 Tropism and Model Virus Infection in Human Cells and Organoids. Cell Stem Cell.

[bib48] Zeberg H., Pääbo S. (2020). The major genetic risk factor for severe COVID-19 is inherited from Neanderthals. Nature.

[bib49] Zhang Q., Raoof M., Chen Y., Sumi Y., Sursal T., Junger W., Brohi K., Itagaki K., Hauser C.J. (2010). Circulating mitochondrial DAMPs cause inflammatory responses to injury. Nature.

[bib50] Zhou T., Tan L., Cederquist G.Y., Fan Y., Hartley B.J., Mukherjee S., Tomishima M., Brennand K.J., Zhang Q., Schwartz R.E. (2017). High-Content Screening in hPSC-Neural Progenitors Identifies Drug Candidates that Inhibit Zika Virus Infection in Fetal-like Organoids and Adult Brain. Cell Stem Cell.

[bib51] Zhou Z., Ren L., Zhang L., Zhong J., Xiao Y., Jia Z., Guo L., Yang J., Wang C., Jiang S. (2020). Heightened Innate Immune Responses in the Respiratory Tract of COVID-19 Patients. Cell Host Microbe.

[bib52] Ziegler C.G., Allon S.J., Nyquist S.K., Mbano I.M., Miao V.N., Tzouanas C.N., Cao Y., Yousif A.S., Bals J., Hauser B.M. (2020). SARS-CoV-2 Receptor ACE2 Is an Interferon-Stimulated Gene in Human Airway Epithelial Cells and Is Detected in Specific Cell Subsets across Tissues. Cell.

[bib53] Zong S., Wu M., Gu J., Liu T., Guo R., Yang M. (2018). Structure of the intact 14-subunit human cytochrome c oxidase. Cell Res..

