# Supplementary material for: A human iPSC-array-based GWAS identifies a virus susceptibility locus in the NDUFA4 gene and functional variants
Source: Cell Stem Cell. 2022 Oct 6;29(10):1475–1490.e6. doi: 10.1016/j.stem.2022.09.008 (PMC9550219; doi:10.1016/j.stem.2022.09.008)
Supplement: Document S1. Figures S1–S7 and Tables S1 and S3–S7 [file mmc1.pdf]

Cell Stem Cell, Volume *29*

Supplemental Information

A human iPSC-array-based GWAS identifies a virus susceptibility locus in the NDUFA4 gene and functional variants

Yuling Han, Lei Tan, Ting Zhou, Liuliu Yang, Lucia Carrau, Lauretta A. Lacko, Mohsan Saeed, Jiajun Zhu, Zeping Zhao, Benjamin E. Nilsson-Payant, Filipe Tenorio Lira Neto, Clare Cahir, Alice Maria Giani, Jin Chou Chai, Yang Li, Xue Dong, Dorota Moroziewicz, The NYSCF Global Stem Cell Array Team, Daniel Paull, Tuo Zhang, Soyeon Koo, Christina Tan, Ron Danziger, Qian Ba, Lingling Feng, Zhengming Chen, Aaron Zhong, Gilbert J. Wise, Jenny Z. Xiang, Hui Wang, Robert E. Schwartz, Benjamin R. tenOever, Scott A. Noggle, Charles M. Rice, Qibin Qi, Todd Evans, and Shuibing Chen

Figure S1

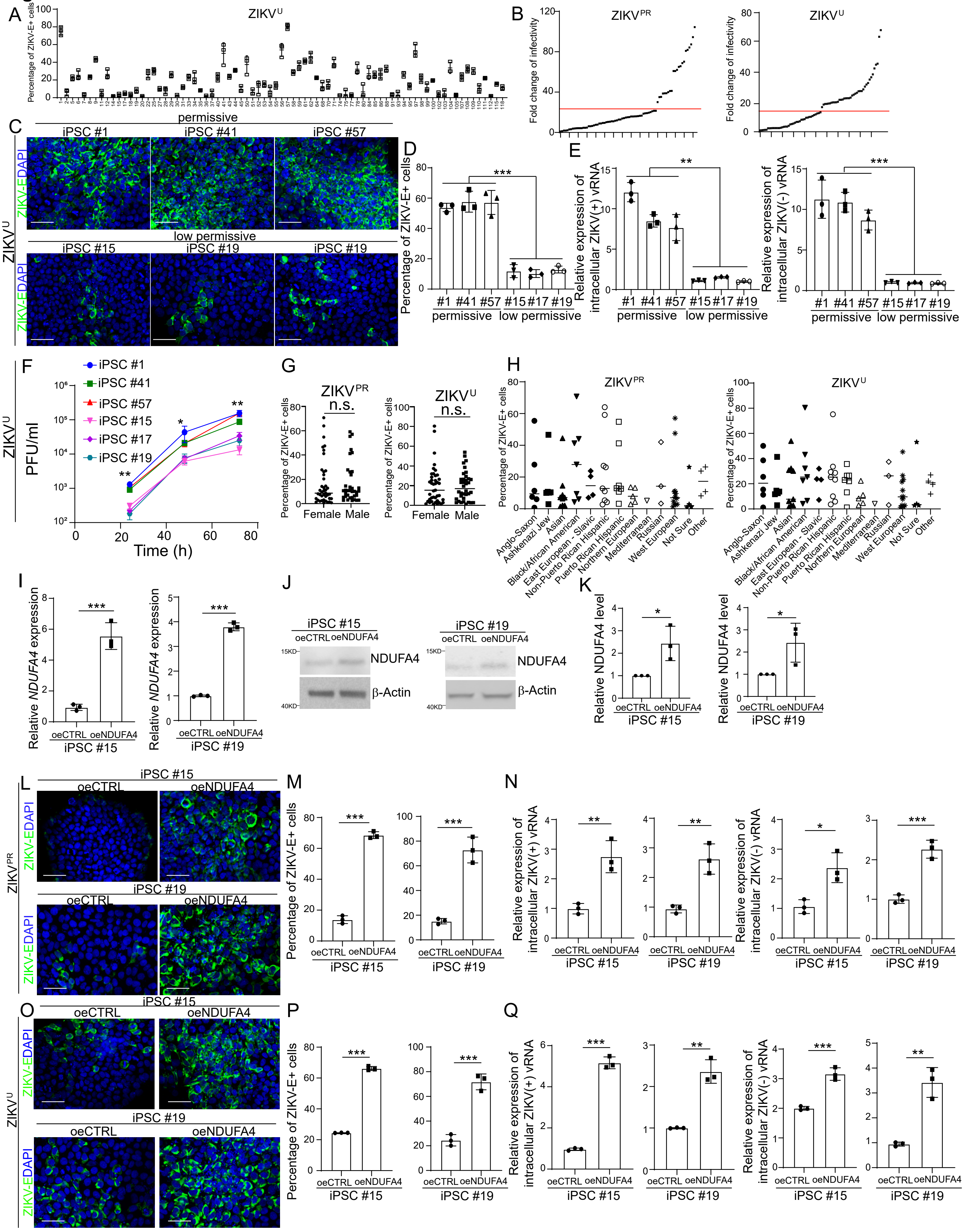

A
ZIKV^U
Percentage of ZIKV-E+ cells
B
ZIKV^PR
ZIKV^U
Fold change of infectivity
C
permissive
iPSC #1
iPSC #41
iPSC #57
low permissive
iPSC #15
iPSC #19
iPSC #19
ZIKV-E DAPI
ZIKV^U
D
Percentage of ZIKV-E+ cells
#1 #41 #57 #15 #17 #19
permissive low permissive
E
Relative expression of intracellular ZIKV(+) vRNA
Relative expression of intracellular ZIKV(-) vRNA
F
iPSC #1
iPSC #41
iPSC #57
iPSC #15
iPSC #17
iPSC #19
PFU/ml
Time (h)
G
ZIKV^PR
ZIKV^U
n.s.
Female Male
H
Anglo-Saxon
Ashkenazi Jew
Asian
Black/African American
East European - Slavic
Non-Puerto Rican Hispanic
Puerto Rican Hispanic
Northern European
Mediterranean
Russian
West European
Not Sure
Other
I
Relative NDUFA4 expression
oeCTRL oeNDUFA4
iPSC #15
iPSC #19
J
NDUFA4
β-Actin
15KD
40KD
K
Relative NDUFA4 level
L
oeCTRL
oeNDUFA4
ZIKV^PR
M
N
O
P
Q

SUPPLEMENTAL FIGURES

Figure S1, related to Figure 1. Primary data of iPSC screening.

(A) The percentage of ZIKV-E positive cells of all iPSC lines upon $ZIKV^{U}$ infection ($ZIKV^{U}$, MOI=0.25). Three biological replicates were used to calculate the infectivity (each replicate includes one well of a 96-well plate, and biological replicates were performed on different days).

(B) The curve trends of infectivity of iPSC lines at 72 hpi ($ZIKV^{PR}$, MOI=1; $ZIKV^{U}$, MOI=0.25).

(C and **D)** Representative confocal images (C) and the quantification (D) of ZIKV-E staining of permissive cell lines: iPSC #1, iPSC #41 and iPSC #57 or low permissive cell lines: iPSC #15, iPSC #17 and iPSC #19 at 72 hpi ($ZIKV^{U}$, MOI=0.15). Scale bar=50 μm.

(E) qRT-PCR analysis of (+) and (-) ZIKV vRNA strands in permissive cell lines: iPSC #1, iPSC #41 and iPSC #57 or low permissive cell lines: iPSC #15, iPSC #17 and iPSC #19 at 72 hpi ($ZIKV^{U}$, MOI=0.15). The value was normalized to *ACTB*.

(F) Multiple step growth curve of ZIKV in the supernatant of permissive cell lines: iPSC #1, iPSC #41 and iPSC #57 or low permissive cell lines: iPSC #15, iPSC #17 and iPSC #19 ($ZIKV^{U}$, MOI=0.15).

(G) Comparison of the infection rate between two groups of iPSC lines derived from female or male donors.

(H) Comparison of the infection rate among groups of iPSC lines derived from distinct ethnic groups.

(I) qRT-PCR analysis of *NDUFA4* mRNA expression levels in iPSC #15-oeCTRL, iPSC #15-oeNDUFA4 hiPSCs or iPSC #19-oeCTRL, iPSC #19-oeNDUFA4 hiPSCs. The value was normalized to *ACTB*.

(J and K) Western blotting and quantification analysis of NDUFA4 protein expression levels in iPSC #15-oeCTRL, iPSC #15-oeNDUFA4 hiPSCs or iPSC #19-oeCTRL, iPSC #19-oeNDUFA4 hiPSCs. β-Actin was used as a loading control.

(L and **M)** Representative confocal images (L) and the quantification (M) of ZIKV-E staining in iPSC #15-oeCTRL, iPSC #15-oeNDUFA4 hiPSCs or iPSC #19-oeCTRL, iPSC #19-oeNDUFA4 hiPSCs at 72 hpi ($ZIKV^{PR}$, MOI=1). Scale bar=50 μm.

(N) qRT-PCR analysis of (+) and (-) ZIKV vRNA strands in iPSC #15-oeCTRL, iPSC #15-oeNDUFA4 hiPSCs or iPSC #19-oeCTRL, iPSC #19-oeNDUFA4 hiPSCs at 72 hpi ($ZIKV^{PR}$, MOI=1). The value was normalized to *ACTB*.

(O and **P)** Representative confocal images (O) and the quantification (P) of ZIKV-E staining in iPSC #15-oeCTRL, iPSC #15-oeNDUFA4 hiPSCs or iPSC #19-oeCTRL, iPSC #19-oeNDUFA4 hiPSCs at 72 hpi ($ZIKV^{U}$, MOI=0.15). Scale bar=50 μm.

(Q) qRT-PCR analysis of (+) and (-) ZIKV vRNA strands in iPSC #15-oeCTRL, iPSC #15-oeNDUFA4 hiPSCs or iPSC #19-oeCTRL, iPSC #19-oeNDUFA4 hiPSCs at 72 hpi ($ZIKV^{U}$, MOI=0.15). The value was normalized to *ACTB*.

Data are representative of at least three independent experiments. Data are shown as mean ± SD. *P* values were calculated by unpaired two-tailed Student's t-test; n.s. no significance, *$P <$ 0.05, **$P <$ 0.01, and ***$P <$ 0.001.

Figure S2

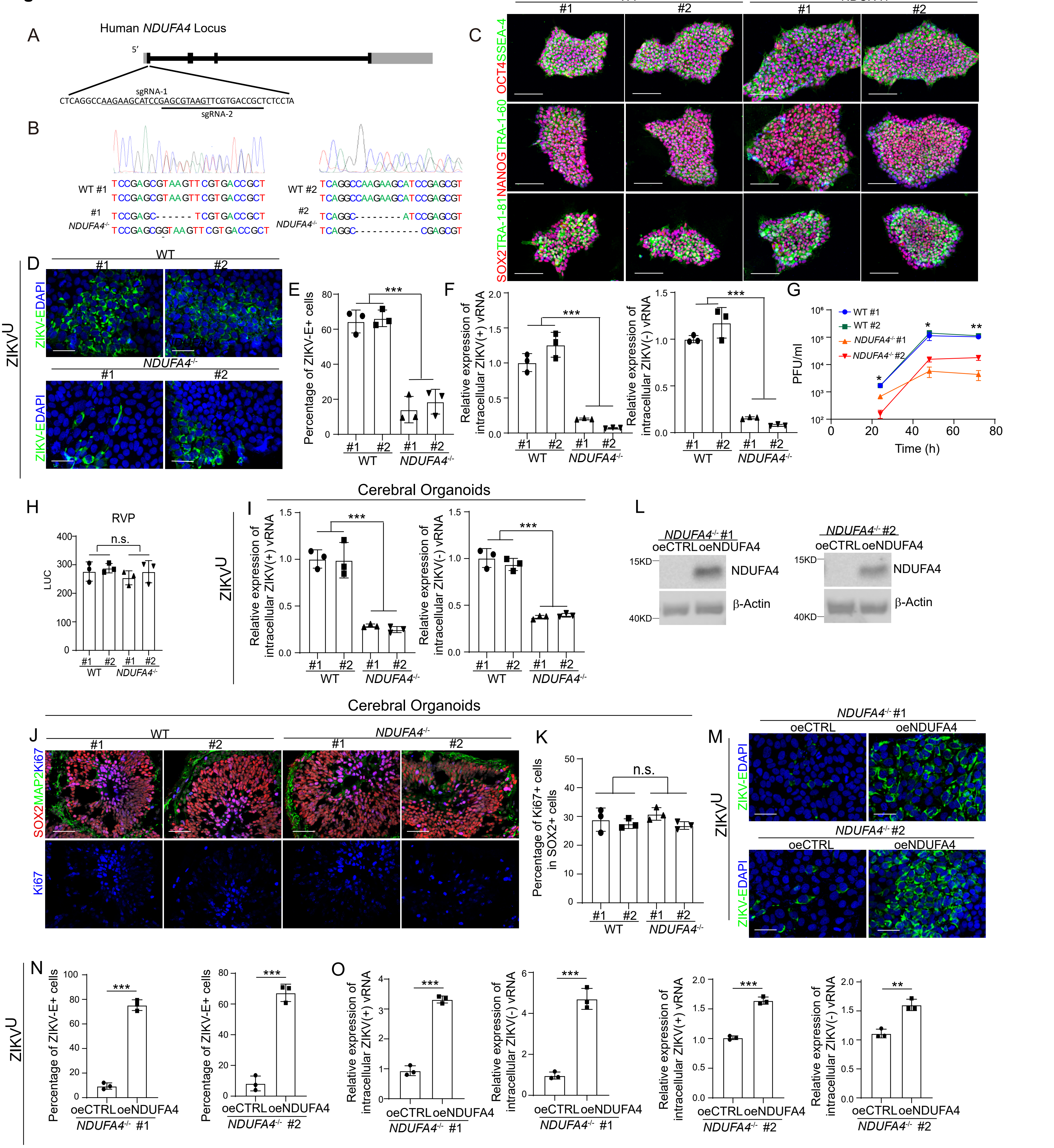

A
Human NDUFA4 Locus
sgRNA-1
CTCAGGCCAAGAAGCATCCGAGCGTAAGTTCGTGACCGCTCTCCTA
sgRNA-2
B
WT #1
#1 NDUFA4-/-
WT #2
#2 NDUFA4-/-
C
WT
NDUFA4-/-
OCT4SSEA-4
NANOGTRA-1-60
SOX2TRA-1-81
D
ZIKV-EDAPI
ZIKVU
E
Percentage of ZIKV-E+ cells
F
Relative expression of intracellular ZIKV(+) vRNA
Relative expression of intracellular ZIKV(-) vRNA
G
PFU/ml
Time (h)
H
RVP
LUC
n.s.
Cerebral Organoids
I
L
oeCTRL
oeNDUFA4
NDUFA4
β-Actin
15KD
40KD
J
SOX2MAP2Ki67
Ki67
K
Percentage of Ki67+ cells in SOX2+ cells
M
N
O

Figure S2, related to Figure 2. *NDUFA4* is associated with ZIKV infection.

(A) Scheme of sgRNA to create *NDUFA4*$^{-/-}$ iPSC lines.

(B) DNA sequencing result of *NDUFA4*$^{-/-}$ #1 and *NDUFA4*$^{-/-}$ #2.

(C) WT and *NDUFA4*$^{-/-}$ iPSCs express pluripotency markers. Scale bar=50 μm.

(D and E) Immunocytochemistry staining (D) and the quantification (E) of ZIKV-E$^+$ cells from WT or *NDUFA4*$^{-/-}$ iPSCs at 72 hpi (ZIKV^U, MOI=0.15). Scale bar=50 μm.

(F) qRT-PCR analysis of (+) and (-) ZIKV vRNA strands in ZIKV infected WT or *NDUFA4*$^{-/-}$ hiPSCs at 72 hpi (ZIKV^U, MOI=0.15). The value was normalized to *ACTB*.

(G) Multiple step growth curve of ZIKV virus in the supernatant of ZIKV infected WT or *NDUFA4*$^{-/-}$ hiPSCs (ZIKV^U, MOI=0.15).

(H) Luciferase activity of WT or *NDUFA4*$^{-/-}$ hiPSCs at 24 hpi with ZIKV RVP.

(I) qRT-PCR analysis of (+) and (-) ZIKV vRNA strands in cerebral organoids derived from WT or *NDUFA4*$^{-/-}$ hiPSCs (ZIKV^U, 5 × 10^5 PFU/ml). Cerebral organoids were age-matched and collected at day 20, then infected with ZIKV for 24 hr. After removal of virus-containing medium, organoids were maintained in organoid medium for an additional 3 days. The value was normalized to *ACTB*.

(J and **K)** Representative images (J) and quantification (K) of Ki67$^+$ cells in SOX2$^+$ cells in cerebral organoids derived from WT or *NDUFA4*$^{-/-}$ hiPSCs. Cerebral organoids were age-matched and collected at day 20. Scale bar=50 μm.

(**L**) Western blotting analysis of the expression levels of NDUFA4 in *NDUFA4*$^{-/-}$-oeCTRL or *NDUFA4*$^{-/-}$-oeNDUFA4 hiPSCs. β-Actin was used as a loading control.

(M and **N)** Representative confocal images (M) and the quantification (N) of ZIKV-E$^+$ cells from *NDUFA4*$^{-/-}$-oeCTRL or *NDUFA4*$^{-/-}$-oeNDUFA4 hiPSCs at 72 hpi (ZIKV^U, MOI=0.15). Scale bar=50 μm.

(O) qRT-PCR analysis of (+) and (-) ZIKV vRNA strands in ZIKV infected *NDUFA4*$^{-/-}$-oeCTRL or *NDUFA4*$^{-/-}$-oeNDUFA4 hiPSCs at 72 hpi (ZIKV^U, MOI=0.15). The value was normalized to *ACTB*.

Data are representative of at least three independent experiments. Data are shown as mean ± SD. For comparison with more than two samples, *P* values were calculated by two-way ANOVA analysis; For comparison with two samples, *P* values were calculated by an unpaired two-tailed Student's t-test; n.s. no significance, $^{**}P < 0.01$, and $^{***}P < 0.001$.

Figure S3

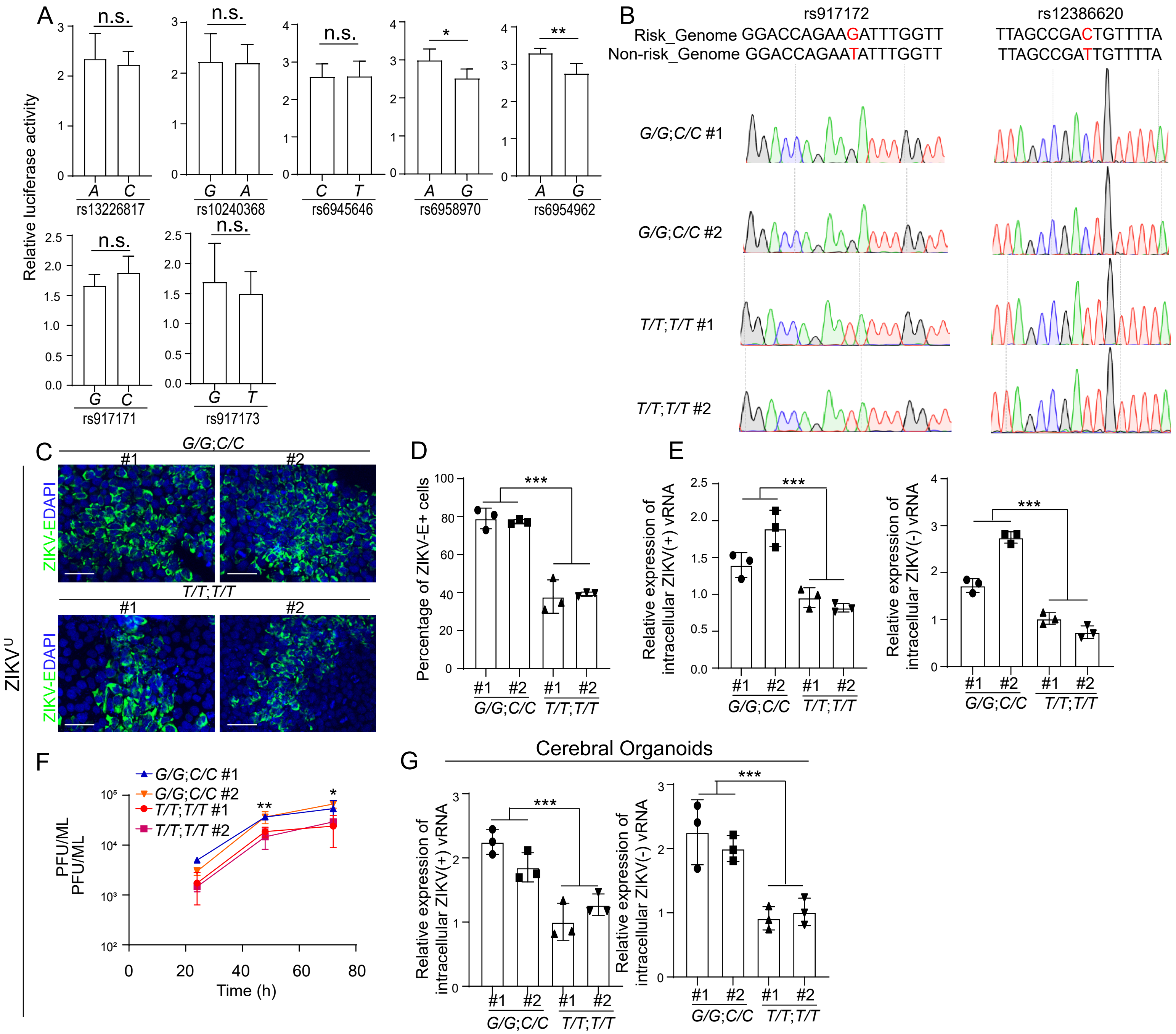
A
Relative luciferase activity
n.s.
rs13226817
rs10240368
rs6945646
rs6958970
rs6954962
rs917171
rs917173
B
rs917172
rs12386620
Risk_Genome GGACCAGAAGATTTGGTT
Non-risk_Genome GGACCAGAATATTTGGTT
TTAGCCGACTGTTTTA
TTAGCCGATTGTTTTA
G/G;C/C #1
G/G;C/C #2
T/T;T/T #1
T/T;T/T #2
C
G/G;C/C
T/T;T/T
ZIKV-EDAPI
ZIKV^U
D
Percentage of ZIKV-E+ cells
E
Relative expression of intracellular ZIKV(+) vRNA
Relative expression of intracellular ZIKV(-) vRNA
F
PFU/ML
Time (h)
G
Cerebral Organoids

Figure S3, related to Figure 3. SNPs of *NDUFA4* promote the infection of ZIKV virus.

(A) Relative luciferase activity of reporters carrying risk/non-risk alleles.

(B) DNA sequencing results of hiPSC lines carrying risk (*G/G*; *C/C*) or non-risk (*T/T*; *T/T*) alleles.

(C and **D)** Representative confocal images (C) and the quantification (D) of ZIKV-E staining in ZIKV infected hiPSC lines carrying risk (*G/G*; *C/C*) or non-risk (*T/T*; *T/T*) alleles at 72 hpi ($ZIKV^{U}$, MOI=0.15). Scale bar=50 μm.

(E) qRT-PCR analysis of (+) and (-) ZIKV vRNA strands in ZIKV infected hiPSC lines carrying risk (*G/G*; *C/C*) or non-risk (*T/T*; *T/T*) alleles at 72 hpi ($ZIKV^{U}$, MOI=0.15). The value was normalized to *ACTB*.

(F) Multiple-step growth curve of ZIKV in the supernatant of ZIKV infected hiPSC lines carrying risk (*G/G*; *C/C*) or non-risk (*T/T*; *T/T*) alleles ($ZIKV^{U}$, MOI=0.15).

(G) qRT-PCR analysis of (+) and (-) ZIKV vRNA strands in cerebral organoids derived from hiPSC lines carrying risk (*G/G*; *C/C*) or non-risk (*T/T*; *T/T*) alleles ($ZIKV^{U}$, 5×10^{5} PFU/ml). Cerebral organoids were age-matched and collected at day 20, then infected with ZIKV for 24 hr. After the removal of virus-containing medium, organoids were maintained in the organoid medium for an additional 3 days. The value was normalized to *ACTB*.

Data are representative of at least three independent experiments. Data are shown as mean ± SD. For comparison with more than two samples, *P* values were calculated by two-way ANOVA analysis; For comparison with two samples, *P* values were calculated by an unpaired two-tailed Student's t-test; $^{*}P < 0.05$, $^{**}P < 0.01$, and $^{***}P < 0.001$.

Figure S4

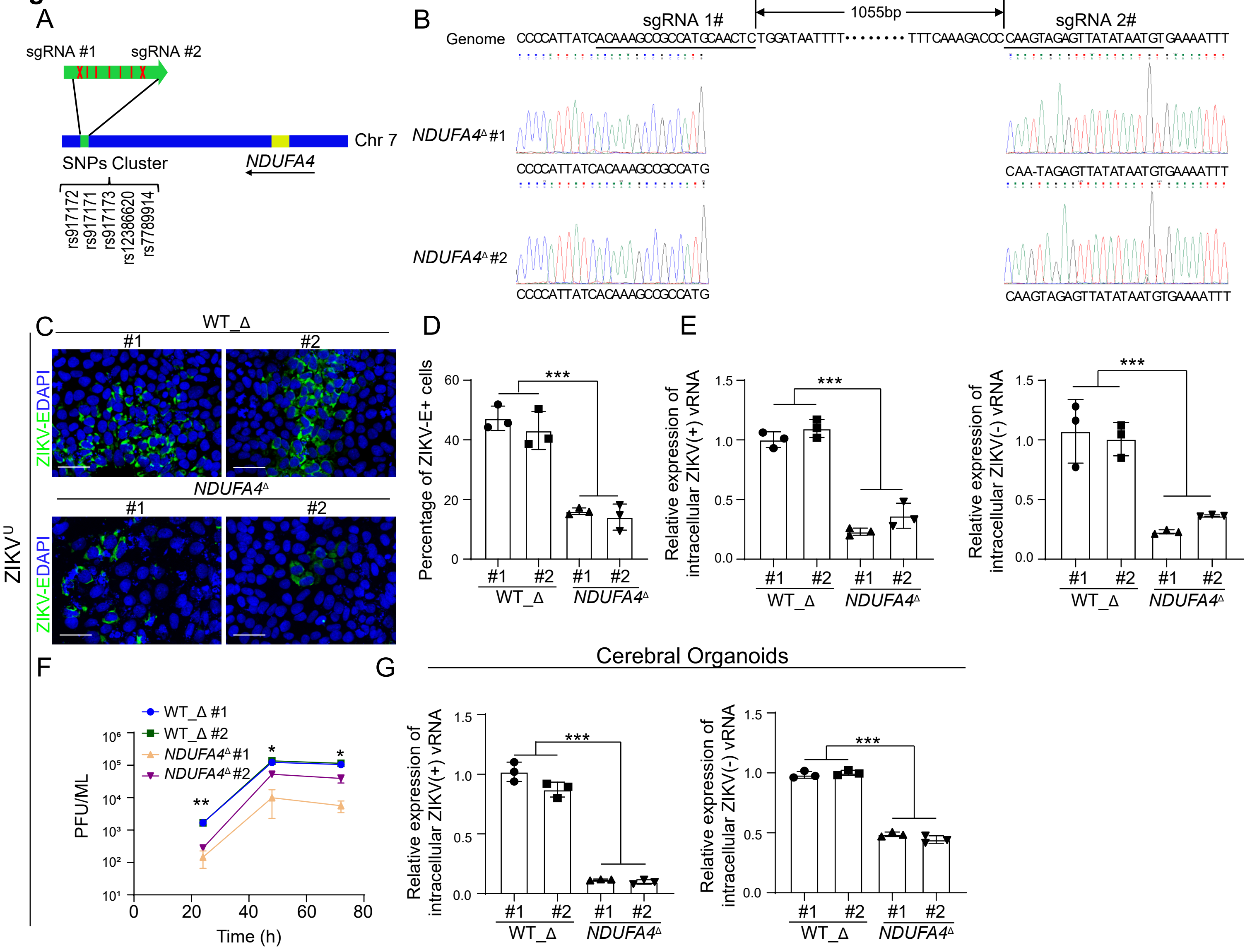
A
sgRNA #1
sgRNA #2
Chr 7
SNPs Cluster
NDUFA4
rs917172
rs917171
rs917173
rs12386620
rs7789914
B
sgRNA 1#
1055bp
sgRNA 2#
Genome
NDUFA4Δ #1
NDUFA4Δ #2
C
WT_Δ
#1
#2
ZIKV-EDAPI
NDUFA4Δ
ZIKVU
D
Percentage of ZIKV-E+ cells
E
Relative expression of intracellular ZIKV(+) vRNA
Relative expression of intracellular ZIKV(-) vRNA
Cerebral Organoids
F
WT_Δ #1
WT_Δ #2
NDUFA4Δ #1
NDUFA4Δ #2
PFU/ML
Time (h)
G

Figure S4, related to Figure 4. Deletion of *cis*-regulatory region decreased the sensitivity to ZIKV infection.

(A) Relative location of the *NDUFA4* gene and a cluster of SNPs.

(B) Sequencing results of WT_Δ and *NDUFA4*$^{\Delta}$ hiPSC lines.

(C and **D)** Representative images (C) and the quantification (D) of ZIKV-E staining in ZIKV infected WT_Δ or *NDUFA4*$^{\Delta}$ hiPSCs at 72 hpi (ZIKV^{U}, MOI=0.15). Scale bar=50 μm.

(E) qRT-PCR analysis of (+) and (-) ZIKV vRNA strands in ZIKV infected WT_Δ or *NDUFA4*$^{\Delta}$ hiPSCs at 72 hpi (ZIKV^{U}, MOI=0.15). The value was normalized to *ACTB*.

(F) Multiple step growth curve of ZIKV virus in the supernatant of ZIKV infected WT_Δ or *NDUFA4*$^{\Delta}$ hiPSCs (ZIKV^{U}, MOI=0.15).

(G) qRT-PCR analysis of (+) and (-) ZIKV vRNA strands of cerebral organoids derived from WT_Δ and *NDUFA4*$^{\Delta}$ hiPSCs (ZIKV^{U}, 5 × 10^5 PFU/ml). Cerebral organoids were age-matched and collected at day 20, then infected with ZIKV for 24 hr. After the removal of virus-containing medium, organoids were maintained in the organoid medium for an additional 3 days. The value was normalized to *ACTB*.

Data are representative of at least three independent experiments. Data are shown as mean ± SD. *P* values were calculated by two-way ANOVA analysis; *P < 0.05, **P < 0.01, and ***P < 0.001.

Figure S5

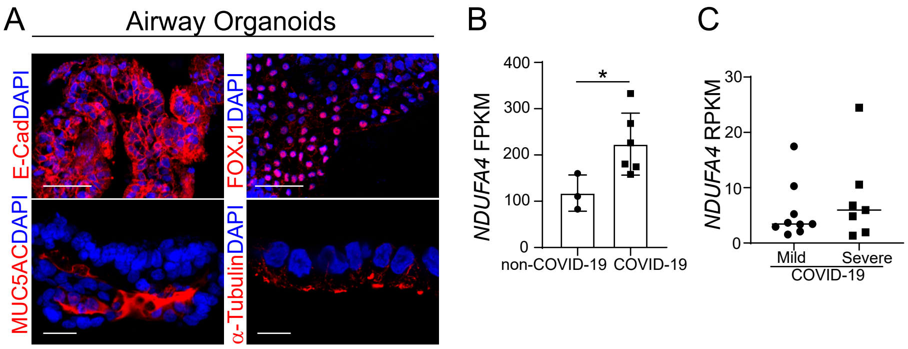

A
Airway Organoids
E-Cad DAPI
FOXJ1 DAPI
MUC5AC DAPI
α-Tubulin DAPI
B
NDUFA4 FPKM
400
300
200
100
0
*
non-COVID-19
COVID-19
C
NDUFA4 RPKM
30
20
10
0
Mild
Severe
COVID-19

Figure S5, related to Figure 5. NDUFA4 expression in COVID-19 patients.

(A) Characterization of human iPSC-derived airway organoids. E-Cad, FOXJ1 and MUC5AC images: Scale bar=50 μm; α-Tubulin image: Scale bar=10 μm.

(B) RNA-seq results of NDUFA4 in COVID-19 patients and non-COVID-19 donors (GSE155241) (Han et al., 2021).

(C) RNA-seq results of NDUFA4 in severe COVID-19 and mild COVID-19 (GSE196822) (Banerjee et al., 2022).

Data are representative of at least three independent experiments. Data are shown as mean ± SD. *P* values were calculated by unpaired two-tailed Student's t-test; $^*P < 0.05$.

Figure S6

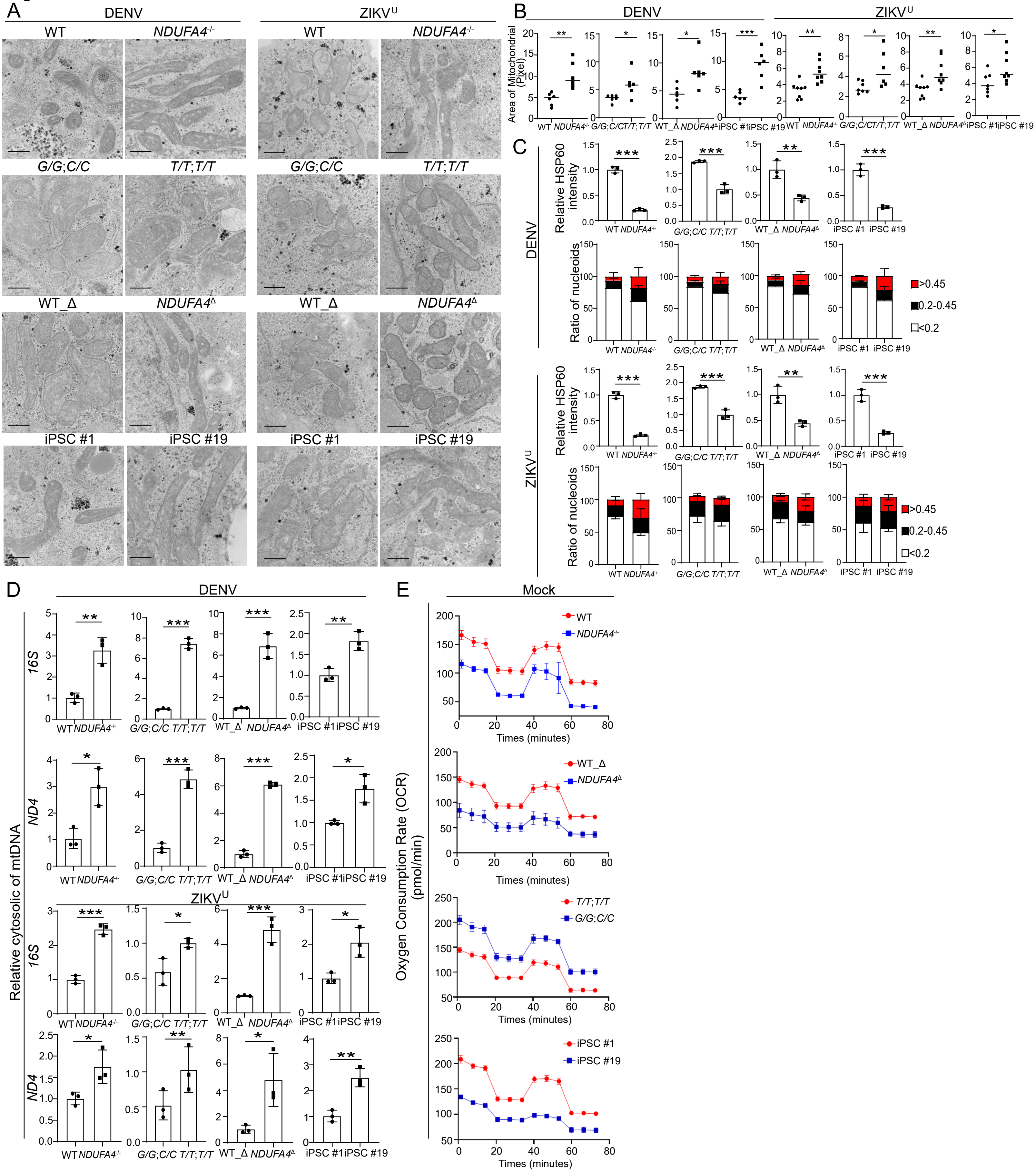
A
DENV
ZIKVᵁ
WT
NDUFA4-/-
G/G;C/C
T/T;T/T
WT_Δ
NDUFA4Δ
iPSC #1
iPSC #19
B
Area of Mitochondrial (Pixel)
C
Relative HSP60 intensity
Ratio of nucleoids
>0.45
0.2-0.45
<0.2
D
Relative cytosolic of mtDNA
16S
ND4
E
Mock
Oxygen Consumption Rate (OCR) (pmol/min)
Times (minutes)

Figure S6, related to Figure 6. Loss or reduction of NDUFA4 causes mitochondrial DNA leaking.

(A and **B)** Representative electron microscopy images (A) and the quantification of mitochondrial size (B) of iPSC lines with DENV or ZIKV infection at 48 hpi (DENV, MOI=1; $ZIKV^{U}$, MOI=0.15). 4 groups: WT v.s. $NDUFA4^{-/-}$; risk (*G/G*; *C/C*) v.s. non-risk (*T/T*; *T/T*); WT_Δ v.s. $NDUFA4^{\Delta}$; and iPSC #1 v.s. iPSC #19. Scale bars= 500 nm.

(C) Quantification of HSP60 staining intensity and nucleoid area in DENV or ZIKV infected conditions (DENV, MOI=1; $ZIKV^{U}$, MOI=0.15).

(D) qPCR analysis of mitochondrial DNA leakage in cytoplasm after DENV or ZIKV infection in hiPSCs at 48 hpi (DENV, MOI=1; $ZIKV^{U}$, MOI=0.15). 4 groups: WT v.s. $NDUFA4^{-/-}$; risk (*G/G*; *C/C*) v.s. non-risk (*T/T*; *T/T*); WT_Δ v.s. $NDUFA4^{\Delta}$; and iPSC #1 v.s. iPSC #19.

(E) Analysis of oxygen consumption rate (OCR) in 4 groups of hiPSCs. 4 groups: WT v.s. $NDUFA4^{-/-}$; risk (*G/G*; *C/C*) v.s. non-risk (*T/T*; *T/T*); WT_Δ v.s. $NDUFA4^{\Delta}$; and iPSC #1 v.s. iPSC #19.

Data are representative of at least three independent experiments. Data are shown as mean ± SD. *P* values were calculated by an unpaired two-tailed Student's t-test; $*P < 0.05$, $**P < 0.01$, and $***P < 0.001$.

Figure S7

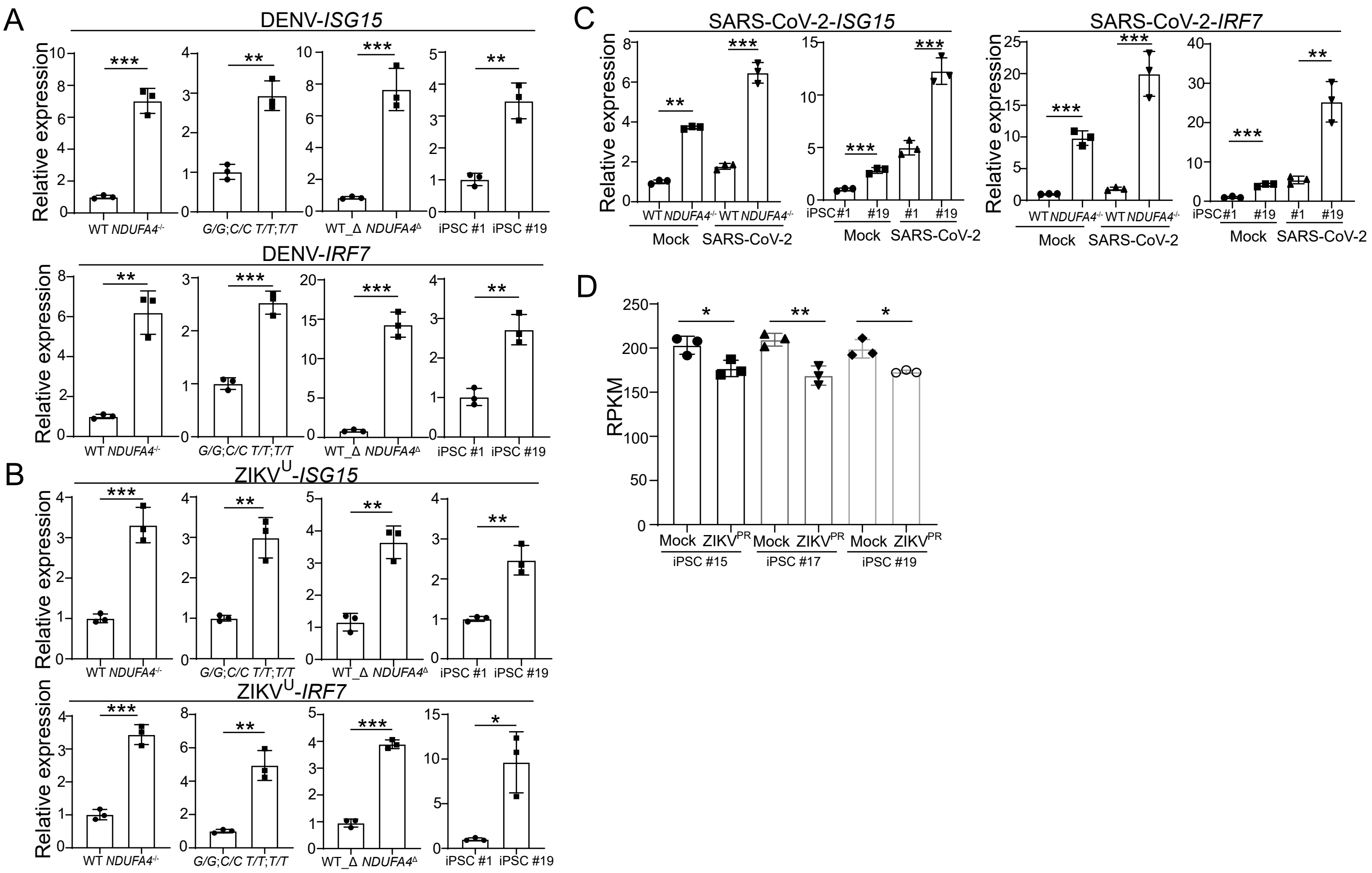
A
DENV-ISG15
DENV-IRF7
Relative expression
WT NDUFA4-/-
G/G;C/C T/T;T/T
WT_Δ NDUFA4Δ
iPSC #1 iPSC #19
B
ZIKVU-ISG15
ZIKVU-IRF7
C
SARS-CoV-2-ISG15
SARS-CoV-2-IRF7
Mock SARS-CoV-2
D
RPKM
Mock ZIKVPR
iPSC #15
iPSC #17
iPSC #19

Figure S7, related to Figure 7. Loss or reduction of NDUFA4 triggers type 1 interferon signaling.

(A) qRT-PCR analysis of *ISG15* and *IRF7* mRNA expression levels with DENV infection at 48 hpi (DENV, MOI=1). 4 groups: WT v.s. $NDUFA4^{-/-}$; risk (*G/G*; *C/C*) v.s. non-risk (*T/T*; *T/T*); WT_Δ v.s. $NDUFA4^{\Delta}$; and iPSC #1 v.s. iPSC #19. The value was normalized to *ACTB*.

(B) qRT-PCR analysis of *ISG15* and *IRF7* mRNA expression levels with ZIKV infection at 48 hpi ($ZIKV^{U}$, MOI=0.15). 4 groups: WT v.s. $NDUFA4^{-/-}$; risk (*G/G*; *C/C*) v.s. non-risk (*T/T*; *T/T*); WT_Δ v.s. $NDUFA4^{\Delta}$; and iPSC #1 v.s. iPSC #19. The value was normalized to *ACTB*.

(C) qRT-PCR analysis of *ISG15* and *IRF7* mRNA expression levels with mock and SARS-CoV-2 infection at 24 hpi (SARS-CoV-2, MOI=0.1) in iPSC-derived airway organoids. 4 groups: WT v.s. $NDUFA4^{-/-}$; risk (*G/G*; *C/C*) v.s. non-risk (*T/T*; *T/T*); WT_Δ v.s. $NDUFA4^{\Delta}$; and iPSC #1 v.s. iPSC #19. The value was normalized to *ACTB*.

(D) RNA sequencing of *NDUFA4* expression levels in low-permissive hiPSC lines with ZIKV infection at 72 hpi ($ZIKV^{PR}$, MOI=1).

Data are representative of at least three independent experiments. Data are shown as mean ± SD. *P* values were calculated by an unpaired two-tailed Student's t-test; *$P < 0.05$, **$P < 0.01$, and ***$P < 0.001$.

Table S1. Information of iPSC array, related to Figure 1.

iPSC line	Gender	Ethnicity
1	F	Hispanic (Not Puerto Rican or Mexican)
2	F	Hispanic (Not Puerto Rican or Mexican)
5	F	Other
6	F	Hispanic (Not Puerto Rican or Mexican)
7	F	Not Sure
8	M	Hispanic (Not Puerto Rican or Mexican)
9	M	Black/African American (Not of Hispanic Origin)
11	F	Black/African American (Not of Hispanic Origin)
12	M	Russian
14	F	Anglo-Saxon
15	M	Russian
17	F	Northern European
18	M	Not Sure
19	F	Asian
20	F	Puerto Rican Hispanic
22	F	Puerto Rican Hispanic
25	F	Asian
27	F	West European
28	F	West European
29	F	Anglo-Saxon | West European
30	F	West European
31	M	Other
33	F	West European
34	F	Northern European
35	F	Northern European
36	M	West European
37	F	Not Sure
40	M	Puerto Rican Hispanic
41	M	Anglo-Saxon
43	F	East European – Slavic | Russian
44	F	Asian
45	M	West European
50	F	Russian
51	F	Asian
52	M	Ashkenazi Jew
53	F	West European
54	M	East European – Slavic

55	M	Ashkenazi Jew
56	M	Asian
57	F	Black/African American (Not of Hispanic Origin)
58	F	Hispanic (Not Puerto Rican or Mexican)
59	F	Puerto Rican Hispanic
61	F	Ashkenazi Jew
62	M	West European
64	F	Black/African American (Not of Hispanic Origin)
68	M	Ashkenazi Jew
70	M	Ashkenazi Jew
71	M	Anglo-Saxon
74	M	West European
75	M	Hispanic (Not Puerto Rican or Mexican)
77	F	Asian
78	F	Black/African American (Not of Hispanic Origin)
80	M	Black/African American (Not of Hispanic Origin)
84	M	Other
85	M	Black/African American (Not of Hispanic Origin)
86	M	Puerto Rican Hispanic
88	F	East European – Slavic
89	F	Anglo-Saxon
92	F	Russian
94	M	West European
95	M	Puerto Rican Hispanic
97	F	Not Sure
98	F	West European
99	F	Anglo-Saxon
100	M	Other
102	F	Northern European
103	M	Mediterranean
104	M	Hispanic (Not Puerto Rican or Mexican)
105	M	West European
107	M	East European – Slavic
108	M	Asian
109	M	Hispanic (Not Puerto Rican or Mexican)
110	F	Hispanic (Not Puerto Rican or Mexican)
111	M	Puerto Rican Hispanic
112	F	Asian | Other
115	F	Anglo-Saxon
118	M	Northern European

Table S2. Information of top 100 SNPs identified from GWAS, related to Figure 1.

Please see the Excel file.

Table S3. RPKM values of coding and non-coding RNAs located in 1Mb upstream or downstream of SNP cluster region, related to Figure 1.

Ensembl Id and Gene name	RPKM ips#1-1	RPKM ips#1-2	RPKM ips#1-3	Location
ENSG00000271526	0	0	0	chr7: 9,084,022-9,084,175
ENSG00000278446	0	0	0	chr7: 9,170,948-9,171,273
ENSG00000233876 *GAPDHP68*	0	0	0	chr7: 9,614,908-9,616,186
ENSG00000271077 *PER4*	0	0	0	chr7: 9,634,270-9,635,817
ENSG00000197320 *LOC340268*	0.596415	0.720182	0.272861	chr7: 9,726,100-9,728,274
ENSG00000234710 *LOC105375148*	0	0	0	chr7: 9,734,270-9,769,513
ENSG00000270611	0	0	0	chr7: 9,850,924-9,851,386
ENSG00000235431 *LOC105375146*	0	0	0	chr7: 9,961,024-9,986,360
ENSG00000212422	0	0	0	chr7: 10,222,797-10,223,011
ENSG00000283117	0	0.016762	0	chr7: 10,449,820-10,779,944
ENSG00000229091 *HSPA8P8*	0	0	0	chr7: 10,451,236-10,453,500
ENSG00000234356 *LOC101154643*	0	0	0	chr7: 10,476,400-10,477,346
ENSG00000236414 *LOC100131472*	0	0	0	chr7: 10,702,672-10,707,538
ENSG00000201774	0	0	0	chr7: 10,901,978-10,902,074
ENSG00000189043 *NDUFA4*	91.89732	86.52564	86.59656	chr7: 10,931,951-10,940,256

Table S4. List of sgRNAs and shRNAs used to create knockout hiPSC lines, related to Figure 2.

Cell line	sgRNA/shRNA
NDUFA4$^{-/-}$*#1*	*ACTTACGCTCGGATGCTTCT*
NDUFA4$^{-/-}$*#2*	*AGCGGTCACGAACTTACGCT*
rs917172-*G/G*	*AGTTCATGGACCAGAATATT*
rs12386620-*C/C*	*GCAGAGAATTATAAAACAAT*
NDUFA4$^{\Delta}$	*ACAAAGCCGCCATGCAACTC*
NDUFA4$^{\Delta}$	*CAAGTAGAGTTATATAATGT*

Table S5. Patient information, related to Figure 3.

Patient ID	Age	Gender	IgG	IgM	RS917172	Rs12386620
5	47	Male	positive	negative	*G*	*C*
6	31	Male	positive	negative	*T*	*T*
8	35	Male	positive	negative	*T*	*T*
11	37	Male	positive	negative	*T*	*T*
19	45	Male	positive	negative	*G*	*C*
20	36	Male	positive	negative	*T*	*T*
21	30	Male	positive	negative	*T*	*T*
22	34	Male	positive	negative	*T*	*T*
23	29	Male	positive	negative	*T*	*T*
26	50	Male	positive	negative	*G*	*C*
28	38	Male	positive	negative	*T/G*	*T/C*
33	37	Male	positive	negative	*T*	*T*
7	30	Male	negative	negative	*G*	*C*
9	35	Male	negative	negative	*T*	*T*
14	32	Male	negative	negative	*T*	*T*
15	32	Male	negative	negative	*T*	*T*
18	40	Male	negative	negative	*T/G*	*T/C*
25	32	Male	negative	negative	*G*	*C*
32	37	Male	negative	negative	*T*	*T*
34	37	Male	negative	negative	*T*	*T*
39	43	Male	negative	negative	*T*	*T*
41	28	Male	negative	negative	*T*	*T*
42	45	Male	negative	negative	*T*	*T*
10	35	Male	borderline	negative	*T*	*T*
35	34	Male	borderline	negative	*T*	*T*

Table S6. Primers/probes used for Sanger sequencing, PCR and qRT-PCR, related to the STAR Methods section.

Primer Name	Sequence
ZIKV (+) vRNA-RT	*TACTTGTACAGCTCGTCCATGCCACTAACGTTCTTTTGCAGACAT*
ZIKV (+) vRNA-PCR forward	*CCGCTGCCCAACACAAG*
ZIKV (+) vRNA-PCR reverse	*TACTTGTACAGCTCGTCCATG*
ZIKV (+) vRNA-PCR probe	*5'-/56-FAM/AGCCTACCT/ZEN/TGACAAGCAATCAGACACTCAA/3IABkFQ/-3'*
ZIKV (-) vRNA-RT	*AACAGCCACAACGTCTATATCCCGCTGCCCAACACAAG*
ZIKV (-) vRNA-PCR forward	*AACAGCCACAACGTCTATATC*
ZIKV (-) vRNA-PCR reverse	*CCACTAACGTTCTTTTGCAGACAT*
ZIKV (-) vRNA-PCR probe	*5'-/56-FAM/TTGAGTGTC/ZEN/TGATTGCTTGTCAAGGTAGGCT/3IABkFQ/-3'*
SARS-CoV-2-TRS-L	*CTCTTGTAGATCTGTTCTCTAAACGAAC*
SARS-CoV-2-TRS-N	*GGTCCACCAAACGTAATGCG*
Human *ACTB*-RT	*CCTGGATAGCAACGTACATGG*
Human *ACTB*-PCR forward	*CCTTGCACATGCCGGAG*
Human *ACTB*-PCR reverse	*ACAGAGCCTCGCCTTTG*
Human *ACTB*-PCR probe	*5'-/5HEX/TCATCCATG/ZEN/GTGAGCTGGCGG/3IABkFQ/-3'*
Human *ACTB*-PCR forward	*ACCTTCTACAATGAGCTGCG*
Human *ACTB*-PCR reverse	*CCTGGATAGCAACGTACATGG*
Human *NDUFA4*-PCR forward	*CATCGGTCAGGCCAAGAA*
Human *NDUFA4*-PCR reverse	*GGGCTCTGGGTTATTTCTGTC*
Human mitochondrial *CYTB* forward	*AACAGCCTTCATAGGCTATGTCCT*
Human mitochondrial *CYTB* reverse	*GGACTGTCTACTGAGTAGCCTCCT*
Human mitochondrial *ND4* forward	*GCTCACACCTCATATCCTCCCTAC*
Human mitochondrial *ND4* reverse	*CAATATTGGCTAAGAGGGAGTGGG*
Human mitochondrial *16S* forward	*CCAACGGAACAAGTTACCCTAGGG*

Human mitochondrial *16S* reverse	*CCGGTCTGAACTCAGATCACGTAG*
Human *ISG15* forward	*CGCAGATCACCCAGAAGATCG*
Human *ISG15* reverse	*TTCGTCGCATTTGTCCACCA*
Human *IRF7* forward	*CCCACGCTATACCATCTACCT*
Human *IRF7* reverse	*GATGTCGTCATAGAGGCTGTTG*
Human nuclear *TERT* forward	*CCCTCCTGTCCAGTTTGCATAAAC*
Human nuclear *TERT* reverse	*TGCTTCCAGACACTTCTCCCCATT*
Human NDUFA4 genotyping forward for coding region	*GGTCGGTTCTCTCCTTTCCA*
Human NDUFA4 genotyping reverse for coding region	*ATGCGGCGAATACAAGAACC*
SNP genotyping forward	*GGGTTCGCGCATTCATTTT*
SNP genotyping reverse	*ACCTTCACCTCCTTCTCTTCT*
rs13226817 forward	*ATACTTCCTAAGAGTCATGCAC*
rs13226817 reverse	*GATTAACTTGTTTGATTCCTTTATTC*
rs917172 forward	*TGGACCAGAAGATTTGGTTTC*
rs917172 reverse	*TGAACTAACTTTGAGTAGC*
rs10240368 forward	*AGATGAAATGGAGTGGTGCAG*
rs10240368 reverse	*TGCAACACAAGACTCCATC*
rs6945646 forward	*TTCTCAAAAACATTCAACCAGAG*
rs6945646 reverse	*CAGTTACCGGTACCTATC*
rs6958970 forward	*CAAAAGGTCAAGAGCTAGCCC*
rs6958970 reverse	*AATTTGATTTTCTTTTCCAAAAACTCAAC*
rs6954962 forward	*AATCCATGAAAAAGCTAGCCC*
rs6954962 reverse	*CAGCCTTCTCAACAGTGTC*
rs917171 forward	*TAGGTACCACGAACATTTGAC*
rs917171 reverse	*TCGATAGAGAAATGTTCTG*
rs917173 forward	*TTGGTCTTGAGTGGCTAGCCC*
rs917173 reverse	*TGAAATTAGAATTTCTGGGTTTAAGGATG*
rs12386620 forward	*AATTAGCCGACTGTTTTATAATTC*
rs12386620 reverse	*TTAATCTTCCTTAATTTTGAGC*
rs2906374 forward	*TTAACTGTTCTTGCTAGTTTATGG*
rs2906374 reverse	*ATGGTAAGACCCGGTAAC*
rs722639 forward	*GAATAAAACAGTTTGTATTCGGTTC*
rs722639 reverse	*AATTTATCAAAGTAGCTTCTG*

Table S7. Antibodies used for immunocytochemistry, intracellular flow cytometry analysis and western blotting analysis, related to the STAR Methods section.

Usage	Antibody	Clone #	Host	Catalog #	Vendor	Dilution
Immunocytochemistry	Anti-DNA	AC-30-10	Mouse	CBL186	Millipore	1:100
Immunocytochemistry	Anti-HSP60	Polyclonal	Rabbit	NBP1-77397	Novus	1:100
Immunocytochemistry	Anti-Flavivirus group antigen	D1-4G2-4-15	Mouse	GTX57154	GeneTex	1:100
Immunocytochemistry	Anti-Flavivirus group antigen	D1-4G2-4-15	Mouse	MAB10216-I-100UG	Millipore	1:1000
Immunocytochemistry & Intracellular flowcytometry	Anti-DENV NS3 protein	Polyclonal	Rabbit	GTX124252	GeneTex	1:500
Immunocytochemistry	Anti-human NANOG	Polyclonal	Goat	AF1997	R & D	1:500
Immunocytochemistry	Anti-OCT-3/4 Antibody	C-10	Mouse	sc-5279	Santa Cruz	1:100
Immunocytochemistry	Anti-SOX2	D6D9	Rabbit	3579	Cell Signaling	1:400
Immunocytochemistry	TRA-1-60 Antibody	TRA-1-60	Mouse	560071	BD Biosciences	1:100
Immunocytochemistry	TRA-1-81 Antibody	TRA-1-81	Mouse	14-8883-82	Thermo Scientific	1:100
Immunocytochemistry & Western blot	Anti-NDUFA4	Polyclonal	Rabbit	ab129752	Abcam	1:200
Immunocytochemistry	Anti-FOXJ1	2A5	Mouse	14-9965-82	Thermo Scientific	1:500
Immunocytochemistry	Anti-Mucin 5AC	45M1	Mouse	ab3649	Abcam	1:100
Immunocytochemistry	Anti-E-Cadherin	4A2	Mouse	14472	Cell Signaling	1:200
Immunocytochemistry	Anti-SARS-CoV/SARS-CoV-2 Nucleocapsid	Monoclonal	Rabbit	40143-R001	SinoBiological	1:1000

Immunocytochemistry	Anti-Acetyl-α-Tubulin (Lys40)	D20G3	Rabbit	5335S	Cell Signaling	1:800
Immunocytochemistry	Anti-MAP2	Polyclonal	Chicken	ab5392	Abcam	1:1000
Immunocytochemistry	Alexa Fluor 594 anti-Mouse IgG (H+L) Secondary Antibody	Polyclonal	Donkey	A-21203	Thermo Scientific	1:500
Immunocytochemistry	Alexa Fluor 488 anti-Rabbit IgG (H+L) Secondary Antibody	Polyclonal	Donkey	A-21206	Thermo Scientific	1:500
Western blot	Anti-β actin	15G5A11/E2	Mouse	MA1-140	Thermo Scientific	1:5000
Western blot	680RD Donkey anti-Mouse IgG Secondary Antibody	Polyclonal	Donkey	926-68072	Li-Cor	1:15000
Western blot	800CW Donkey anti-Rabbit IgG Secondary Antibody	Polyclonal	Donkey	926-32213	Li-Cor	1:15000
